# Biochemistry of Antioxidants: Mechanisms and Pharmaceutical Applications

**DOI:** 10.3390/biomedicines10123051

**Published:** 2022-11-25

**Authors:** Sonia Losada-Barreiro, Zerrin Sezgin-Bayindir, Fátima Paiva-Martins, Carlos Bravo-Díaz

**Affiliations:** 1 Departamento de Química-Física, Facultade de Química, Universidade de Vigo, 36200 Vigo, Spain; 2 Department of Pharmaceutical Technology, Faculty of Pharmacy, Ankara University, 06560 Ankara, Turkey; 3 REQUIMTE-LAQV, Department of Chemistry and Biochemistry, Faculty of Sciences, University of Porto, 4169-007 Porto, Portugal

**Keywords:** oxidative stress, antioxidants, bioavailability, bioactivity, nano antioxidants delivery systems

## Abstract

Natural antioxidants from fruits and vegetables, meats, eggs and fish protect cells from the damage caused by free radicals. They are widely used to reduce food loss and waste, minimizing lipid oxidation, as well as for their effects on health through pharmaceutical preparations. In fact, the use of natural antioxidants is among the main efforts made to relieve the pressure on natural resources and to move towards more sustainable food and pharmaceutical systems. Alternative food waste management approaches include the valorization of by-products as a source of phenolic compounds for functional food formulations. In this review, we will deal with the chemistry of antioxidants, including their molecular structures and reaction mechanisms. The biochemical aspects will also be reviewed, including the effects of acidity and temperature on their partitioning in binary and multiphasic systems. The poor bioavailability of antioxidants remains a huge constraint for clinical applications, and we will briefly describe some delivery systems that provide for enhanced pharmacological action of antioxidants via drug targeting and increased bioavailability. The pharmacological activity of antioxidants can be improved by designing nanotechnology-based formulations, and recent nanoformulations include nanoparticles, polymeric micelles, liposomes/proliposomes, phytosomes and solid lipid nanoparticles, all showing promising outcomes in improving the efficiency and bioavailability of antioxidants. Finally, an overview of the pharmacological effects, therapeutic properties and future choice of antioxidants will be incorporated.

## 1. Introduction—Antioxidants as Pharmacological Agents

Biological processes that take place in the human body, including breathing, metabolizing therapeutic agents, digesting food and converting fats into energy, may produce damaging compounds known as reactive oxygen and nitrogen species, ROS and RNS, collectively named RONs. These reactive species are usually free radicals or species that readily produce free radicals. At low to moderate concentrations, RONs play a key role in different biological routes implicated in several cellular functions. However, high concentrations of RONs may produce lipid, protein and DNA damage, disrupting the normal cellular signaling mechanisms [1].

Under stress conditions, the formation of RONs overwhelms the antioxidant defenses, resulting in a redox imbalance which produces oxidative stress and irreversible alterations in cell compounds [2]. Oxidative stress is the leading factor that produces damage in cell structures, i.e., in membranes, proteins, lipids and DNA. Such damage can compromise cell functions, leading to a variety of cellular responses through the formation of secondary reactive species, affecting the health of cells. It is a key mechanism linked with major chronic diseases including, among others, cancer, cardiovascular, liver and neurological disorders [3].

Antioxidants (AOs) play a vital role in these defense mechanisms [2,3,4]. Cells have an efficient antioxidant system (enzymes and other non-enzymatic molecules) that reduces the adverse impact of free radicals, which are produced continuously by cells. Antioxidants can be effective in the inhibition and/or treatment of chronic disorders, blocking or slowing down the reactions of biomolecules with free radicals by transferring electrons to free radicals and inhibiting the oxidative process.

In general, antioxidant defense comprises different mechanisms. Among these are (1) delaying or inhibiting free radicals production, (2) free radical scavenging, (3) changing free radicals into less toxic compounds, (4) delaying the formation of secondary toxic active species, (5) interrupting the chain propagation reaction (chain breaking antioxidants), (6) boosting the endogenous antioxidant defense system through synergism with other antioxidants and (7) chelating metal ions [5,6].

Supplementation of exogenous natural antioxidants in the diet to improve the endogenous antioxidant defense system of the body is a strategy to reduce the undesirable effects of RONs. However, screening for natural bioantioxidants with suitable pharmacological properties has turned out to be challenging because the activity of natural antioxidants (or natural extracts) in inhibiting oxidative stress depends, among other factors, on their chemical structures, bond dissociation enthalpies and redox potentials and on their effective concentrations at the reaction site with RONs [3].

**Figure 1:**
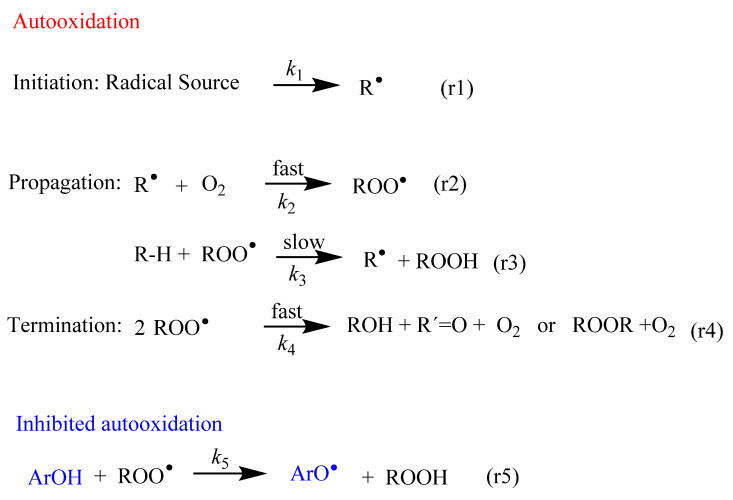
Fundamental steps of the lipid autooxidation and inhibition reactions. The main inhibition mechanism comprises the transfer of an H atom from a phenolic antioxidant (ArOH) to the chain carrying a free radical (ROO^•^). R-H stands for lipid molecule, and R^•^, ROO^•^ and RO^•^ are the derived lipoid, peroxyl and alkoxyl radicals, respectively.

An ideal antioxidant should be readily absorbed, remove RONs and, eventually, chelate metals at physiologically suitable concentrations [7,8]. Phenolic compounds constitute a set of chemical compounds bearing one or more hydroxyl groups (-OH) bonded to an aromatic ring, Figure 1. They present a large variety of bioactivities that depend on the specific chemical structure (number and position of hydroxyl groups and other substituents in the aromatic ring), acting as free radical scavengers, interacting with and diffusing through biological membranes, exerting intracellular signaling effects and binding to receptors and enzymes [7].

For these and other reasons, the pharmacological properties of phenols present in our diet have gained increased attention from researchers in the last several years. However, a direct link between the (strong) antioxidant activity of phenols in vitro and their efficiency in cells or other multiphasic systems is not easy to establish; closer attention needs to be paid to their availability at the reaction site to fully understand their efficiency. One of the possible causes of the lack of antioxidant efficiency is their inability to reach the specific targets at the required concentration. Therefore, a thorough analysis of the mechanisms of action of phenols is necessary to find strategies to improve their bioavailability and bioactivity and to explore their potential use as therapeutic agents to treat multiple pathologies.

## 2. Oxidative Reactions of Free Radicals

In all cells, the formation of free radicals takes place continuously as part of normal cellular function. Figure 1 shows an abbreviated sequence of the lipid autooxidation reaction that takes into account the relative rates of the involved reactions, simplified in terms of the initiation, propagation and termination steps.

In the initiation step, a carbon-centered free radical (R^•^) is produced by H abstraction (or electron transfer) from lipids (reaction 1 in Figure 1). These C-centered free radicals, R^•^, react much faster, at diffusion-control rates, O_2_, leading to the rapid formation of peroxyl radicals ROO^•^ (reaction 2 in Figure 1).

Peroxyl radicals ROO^•^ can react with neighboring lipids, RH, with a rate constant *k*_3_. This reaction is the rate-limiting step of the oxidation reaction and leads to the formation of lipid hydroperoxides, LOOH, and another lipid radical, R^•^. The reaction continues and a variety of products can be obtained through different reaction routes (Table 1) [9,10]. Both peroxyl radicals and hydroperoxides can propagate the oxidative damage in living organisms.

Considering the simplified autooxidation reaction presented in Figure 1 and that the concentration of oxygen is not limiting, the overall rate of propagation *r*_p_ is given by Equation (1).

(1) $$r_{p}=-\frac{d\left[O_{2}\right]}{\mathrm{dt}}=-\frac{d\left[\mathrm{RH}\right]}{\mathrm{dt}}=k_{p}\left[{\mathrm{ROO}}^{•}\right]\left[\mathrm{RH}\right]$$

Peroxyl radicals (ROO^•^) and alkoxyl radicals (RO^•^) may also undergo cyclization to epoxides/rearrangement, disproportionation (ROO^•^ + ROO^•^), addition to double bonds or suffer elimination or scission reactions, competing with hydrogen abstraction and thus making the overall lipid oxidation process very difficult to study. For example, studies show that products other than hydroperoxides can be obtained in the early and end stages of oxidative reactions without going through hydroperoxides [9].

In the termination step, the oxidation reaction terminates through the formation of non-radical products (reaction 4 in Figure 1).

It is worth pointing out that different attempts have been made to model the lipid oxidation mechanism in multiphasic systems, such as emulsions, by fitting the obtained lipid oxidation kinetics to mathematical equations [12]. The exact mechanism of the oxidative reactions, however, depends on the environment where the reaction takes place and on the physicochemical properties of the reactants [10]. Thus, new alternative reaction pathways, not previously considered, may need to be taken into account. In summary, the antioxidant efficiency depends on the particular rates of all the reactions where antioxidants participate, so that a detailed modeling of lipid oxidation becomes a formidable task.

Scavenging of peroxyl radicals (ROO^•^) or repairing of the damaged target molecules can be achieved by enhancing the levels of endogenous antioxidants and/or addition of exogenous antioxidants such as (poly)phenolic compounds or ArO-H (reaction 5 in Figure 1). Polyphenols have the outstanding characteristic of inhibiting or delaying the autoxidation of unsaturated lipid molecules through mechanisms that will be described in Section 4.

## 3. Antioxidants

The human organism has developed specific and complex antioxidant mechanisms (enzymatic and non-enzymatic) which may act synergistically to protect cells and organs against RON damage. Endogenous AOs may not be sufficient to avoid oxidative stress and, to maintain cellular functions, dietary exogenous antioxidants—AOs included in our diet or as dietary supplements—may be needed [13,14,15].

### 3.1. Endogenous Antioxidant Enzymes

Some enzymes can directly inhibit the formation of free radicals (primary enzymes) or can do so indirectly by supporting other endogenous AOs. Some efficient AO enzymes are glutathione peroxidase (Gpx), catalase (CAT) and superoxide dismutase (SOD) [16].

#### 3.1.1. Glutathione Peroxidase (Gpx)

**Figure 2:**
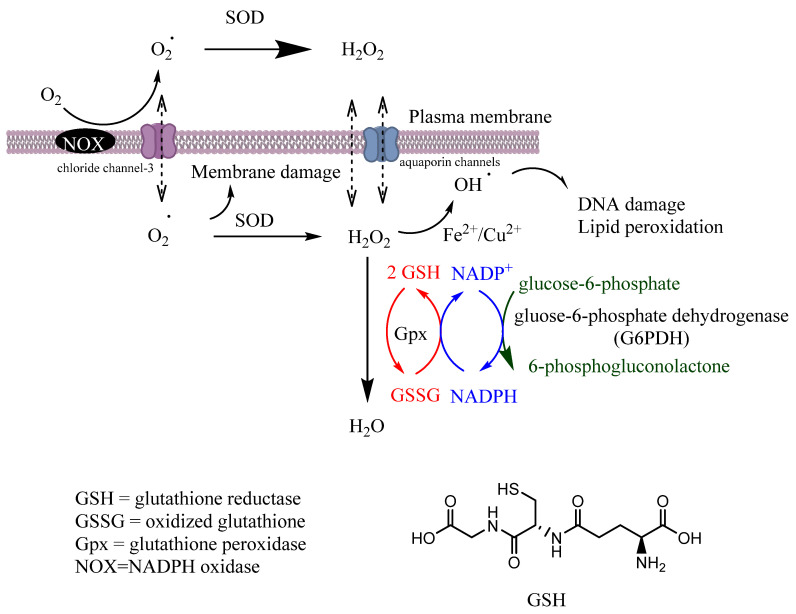
Scavenging of ROS. Coordination between various intracellular antioxidant enzymes (SOD, CAT, GR, Gpx) is a crucial mechanism for controlling ROS homeostasis and signaling. Secondary enzymes Gpx and G6PDH sustain the activity of primary enzymes by regenerating NADPH.

Gpx is a tetrameric selenoprotein that catalyzes the detoxification of H_2_O_2_ (reaction 6) and lipid peroxides (reaction 7) by reducing glutathione (GSH), mainly in the mitochondria but also in the cytosol, playing a key role in the protection of cells from oxidative damage by inhibiting lipid oxidation. In this process, GSH is oxidized to glutathione disulfide (GSSG), that can then be reduced back to GSH by glutathione reductase (GR) in the presence of nicotinamide adenine dinucleotide phosphate (NADPH), Figure 2.

$$\begin{array}{l} H_{2}O_{2}+2\mathrm{GSH}\overset{\mathrm{Gpx}}{\to }{2H}_{2}O+\mathrm{GSSG}\;(r6)\\ \mathrm{ROOH}+2\mathrm{GSH}\overset{\mathrm{Gpx}}{\to }{\mathrm{ROH}+\mathrm{GSSG}+H}_{2}O\;(r7) \end{array}$$

#### 3.1.2. Catalase (CAT)

Catalase (CAT), a tetrameric heme protein, can be found in peroxisomes of aerobic cells and catalyzes the dismutation of hydrogen peroxide to water and molecular oxygen (reaction 8).

$${2H}_{2}O_{2}\overset{\mathrm{CAT}}{\to }{2H}_{2}{O+O}_{2}\;(r8)$$

Interestingly, CAT is one of the enzymes with the highest known rate constant (*k*_CAT_ > 10^6^ s^−1^), close to those of diffusion-controlled reactions [17]. CAT efficiency has been related with various disorder conditions [16].

#### 3.1.3. Superoxide Dismutase (SODs)

Superoxide dismutase (SODs) is vital to cellular health, protecting cells from oxidative damage. It is one of the most powerful intracellular antioxidants, and it catalyzes the dismutation of superoxide anions (O_2_^•−^) to hydrogen peroxide (H_2_O_2_) and molecular oxygen (O_2_), reaction 9 and Figure 2. Its activity needs different metallic cofactors (Fe, Zn, Cu and Mn) depending on the isoform, with specific subcellular distribution. For example, the dimeric copper–zinc SOD (SDO1) is present in cytosol, the manganese–SOD (SDO2) is mitochondrial and the tetrameric copper–zinc SOD (SOD3) is present in plasma (extracellular environment) [17]. SOD deficiency increases with age, suggesting that a proper daily SOD supplementation may protect the immune system [16].

$$2O_{2}^{-}{}^{\cdot }+{2H}^{+}\overset{\mathrm{SOD}}{\to }H_{2}O_{2}{+O}_{2}\;(r9)$$

### 3.2. Non-Enzymatic Endogenous Antioxidants

Non-enzymatic antioxidants can be classified into endogenous (if cells are able to produce them) and exogenous (if antioxidants need to be ingested through the diet (see Section 3.3)).

Some proteins with thiol or phenolic groups have antioxidant properties and can easily scavenge RONs. As an example, glutathione is the most important intracellular thiol, with several antioxidant properties. It is the cofactor of glutathione peroxidase (Gpx) enzymes, has chelating properties and is involved in the recycling of vitamins C and E.

Melatonin (N-acetyl-5-methoxytyptamine), a hormone released by the pineal gland in the brain and involved in circadian rhythm, also has a direct free radical scavenging capacity. In addition, it is able to stimulate several antioxidative enzymes and increase the efficiency of mitochondrial oxidative phosphorylation, reducing electron leakage (thereby lowering free radical generation) [17].

Coenzyme Q10 is another antioxidant present in all respiring eukaryotic cells, primarily in the mitochondria as a component of the electron transport chain. It is a natural lipophilic AO and it is able to inhibit lipid oxidation and regenerate other oxidized AOs, such as vitamins C and E [18].

### 3.3. Exogenous Non-Enzymatic AOs—Dietary Sources and Classification

The postprandial state is one of the major contributors to chronic diseases, because this period is characterized by intense metabolic traffic, biosynthesis and oxidative metabolism of absorbed nutrients, glycerides, lipids, proteins and other constituents of the diet [19,20]. During this state, biological systems activate compensatory and adaptive mechanisms in order to counterbalance the disturbance caused and restore balance/homeostasis. In a young and healthy organism, this imbalance is limited, with a rapid recovery of the system causing minimal stress. This metabolic imbalance occurs several times a day, whenever food is ingested, and at this time there is a great opportunity for cell injury, which, with the aging of the biological defense and repair systems, ends up manifesting in cell dysfunction and disease [19].

**Figure 3:**
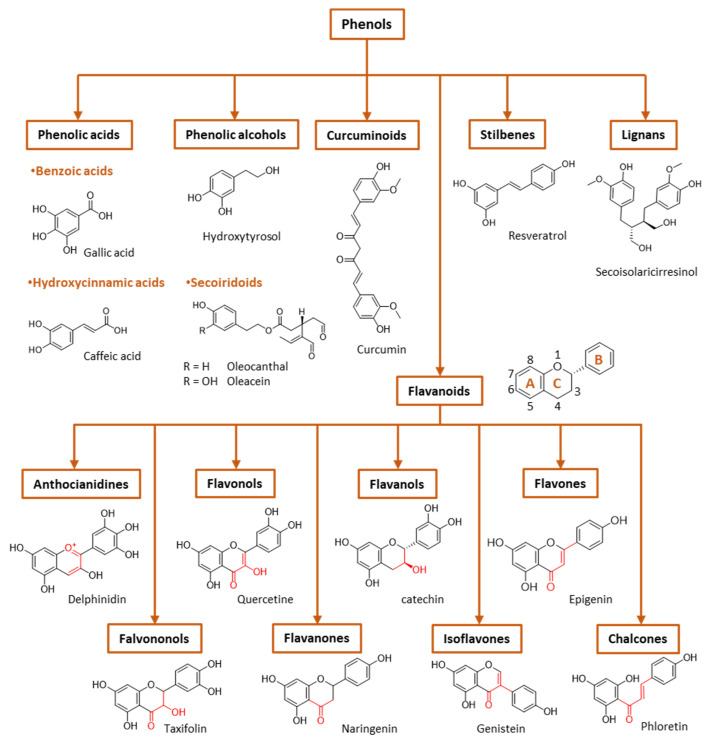
General classification of polyphenols.

During the postprandial state, the imbalance between the production of RONs and their quenching by the body’s antioxidant defenses leads to biological dysfunction and/or cellular damage. When antioxidant systems are overloaded, an excess of RONs species alter the redox balance, increasing activation of pro-inflammatory pathways (e.g., NF-kB) and impairing cell signaling (e.g., insulin signaling) associated with the exacerbation of a number of chronic diseases, such as diabetes and atherosclerotic cardiovascular diseases, metabolic syndrome, hypertension and obesity, among others [21]. A high consumption of carbohydrates and lipids during meals normally induces relatively high (and prolonged) oxidative stress, especially in obese or diabetic individuals, emphasizing the importance of diet in insulin sensitivity and health in general [21]. Moreover, hyperglycemia can promote non-enzymatic glycation of proteins, such as LDL [22], and increase the susceptibility of LDL to oxidation, which, once oxidized, can initiate or facilitate the progression of atherosclerosis [23]. Although the postprandial period causes oxidative stress in the body, this may also be an opportunity for the ingestion of protective substances and intervention, favoring a more balanced redox status. In fact, in the diet, together with nutrients, other substances are ingested that may help to counterbalance these oxidative and inflammatory effects caused by food consumption. Thus, the concomitant consumption of phenol-rich fruits and vegetables in meals may have several advantages in the postprandial state, given their antioxidant activity and potential to modulate cellular redox–oxidative (redox) balance [20]. Phenolic compounds are distributed throughout the plant kingdom and comprise both simple molecules, such as phenolic acids, and complex polymeric compounds, such as tannins and lignans. The most frequent polyphenol classes found in the human diet are flavonoids and phenolic acids (Figure 3), and many studies have shown that these phenols can modulate various biological processes related to the risk of diseases induced by oxidative stress, platelet dysfunction [24], inflammation [25] and initiation and progression of cancer [26]. In fact, the phenolic compounds existing in these foods, being bioavailable, have been shown to increase the plasma antioxidant capacity, both in the short term, when ingested occasionally, and in a sustainable way through frequent consumption [27].

For this reason, there is a substantial upsurge in the number of studies linked to the application of AOs from natural sources. In recent decades, therapies involving plant-derived polyphenols appear to be an attractive approach to inhibiting oxidative damage. Plants have been utilized since ancient times in traditional medicine and for many other applications due to their ability to biosynthesize a wide range of antioxidants capable of attenuating RONs. The interest in natural extracts has increased in the pharmaceutical, cosmetic and food industries due to their bioactive properties, claimed by the quickly growing market of antioxidant-containing healthy foods.

Phenols are the most common class of plant secondary metabolites. In some plants, their concentration can be as high as 750 mg/100 g of fruit [28]. Vegetables and fruits (cereals, dried legumes, nuts, tomatoes, spinach, pears, beans, cinnamon, cherries, oranges, apples) and beverages (red wine, beer, tea, coffee, etc.) are the most widespread rich dietary sources of polyphenols. The presence of polyphenols partially contributes to their sensory and nutritional quality (color, astringency, odor, etc.).

Natural antioxidants can be classified in different ways. Based on their mechanism of action, antioxidants can be classified as primary antioxidants and secondary antioxidants. Another way of classifying antioxidants is based on their solubility in the aqueous or lipid phases. On this basis, the antioxidants can be classified as water-soluble (e.g., hydroxytyrosol, caffeic acid, chlorogenic acid, etc.) and lipid-soluble (e.g., tocopherol, oleocanthal, etc.) antioxidants [28].

Phenolic compounds are the major plant-compounds known to have antioxidant activity, and they show a variety of structures, from relatively simple molecules (e.g., gallic acid, caffeic acid, hydroxytyrosol, etc.) to complex polyphenols such as tannins and flavonoids, Figure 3. Plant phenols are aromatic secondary metabolites that are derived from the shikimate pathway and phenylpropanoid metabolism. Phenolic compounds can be found as free forms or in conjugated forms, attached to one or more sugar residues at the hydroxyl groups or to carboxylic acids, amines, fatty acids or other phenols.

Phenolic compounds are usually classified on the basis of their chemical structure, reflected in the number and position of substituents in the aromatic rings, Figure 3.

(I) Phenolic acids are phenols that contain a carboxylic group directly linked either to the aromatic ring (hydroxybenzoic acids, C1-C6 backbone) or linked to an alkyl group (hydroxycinnamic acids, C3-C6 backbone). Changes in the nature of the substituents and their position in the chemical structure led to a diversity of antioxidants. Figure 3 shows the chemical structures for the most common hydroxybenzoic acid and hydroxycinnamic acid.

- Hydroxybenzoic acids include protocatechuic, vanillic and gallic acids, among others. They frequently can be found glycosylated, bound to small organic acids such as quinic, maleic or tartaric acids, or linked to structural components of plant cells (cellulose, proteins). Hydrolyzable tannins (gallotannins, ellagitannins) can be formed from gallic acid and carbohydrates.
- The most abundant hydroxycinnamic acids are caffeic, p-coumaric and ferulic acids, along with ester derivatives of caffeic acid such as chlorogenic acid.

(II) Flavonoids are the most abundant plant polyphenols, comprising approximately 10,000 natural compounds. They share a phenyl benzopyran structure: two benzene rings (denoted as A and B rings, Figure 3) linked by a heterocyclic pyran ring (ring C). Flavonoids can be classified into eight groups according to the structure of the pyran ring: flavones, flavanols, flavonols, isoflavonoids, flavanones, flavanonols, anthocyanins and chalcones. This classification is based on the structure of the pyranic ring (Figure 3). Flavonoids can quench harmful ROS such as superoxide anion, peroxyl and hydroxyl radicals and chelate several metal ions, including iron and copper [17].

- Flavones are characterized by the presence of a double bond between carbon 2 and 3, a keto group in carbon 4 and a B ring linked to carbon 2. The most common flavones include apigenin, luteolin and their glycosides.
- Isoflavonoids are flavones in which the B ring is linked to carbon 3 instead of carbon 2. This change causes isoflavonoids to resemble estrogens which are well-known as phytoestrogens, presenting a possible mild estrogenic activity. The most abundant isoflavones are glycitein and daidzein, among others, and their 7-O glycosides.
- Flavonols are flavones that have a hydroxyl group at carbon 3. Examples of flavonols are quercetin, myricetin, kaempferol and their glycosides.
- In flavanones and flavononols, ring C is a saturated pyrane ring with a ketone group in carbon 4. Flavononols also contain a –OH group in carbon atom 3. They are not the most abundant flavonoids in nature, but the most common flavonones are naringenin, hesperetin and eriodictyol, and the most common flavononols are taxifolin and its glycosides.
- Flavanols hold a ketone group at carbon 4 and a hydroxyl group at position 3. The most important flavanols are epicatechin, catechin, pirogallocatechin, gallocatechin and their oligomers, polymers and 3-O-gallates.
- Anthocyanidins are ionic flavonoids and differ from the other flavonoids by possessing an –OH group in carbon 3 and two double bonds in ring C. They are the major group of hydrophilic plant pigments, and their color changes with the pH. The most common are malvidin, peonidin, cyaniding, etc., which provide different colors to vegetables.
- Chalcones are characterized by the absence of the C ring of the basic flavonoid skeleton structure and are referred to as open-chain flavonoids. Major examples of chalcones include phloridzin, arbutin, phloretin and chalconaringenin. Chalcones have a common chemical scaffold of 1,3-diaryl-2-propen-1-one (α-, β- unsaturated ketones, also known as chalconoid) that exists as *trans* and *cis* isomers, with the *trans* isomer being thermodynamically more stable.

(III) Stilbenes have a C6–C2–C6 chemical structure with a double bond between the phenolic rings (diarylethene). Resveratrol, piceatannol, pterostilbene, pinosylvin and clomiphene are some examples of stilbene compounds.

(IV) Lignans are formed by the association of two cinnamic acid moieties (C6–C3–C3–C6 skeleton). An example of a lignan is the secoisolariciresinol diglucoside (Figure 3). Lignans can be found in all the organs of many vascular plants but in low concentrations.

(V) Curcuminoids are a relatively small class of phenols that include curcumin, demethoxycurcumin and bisdemethoxycurcumin, all isolated from turmeric (*Curcuma longa*). They have, as a common chemical scaffold, a linear diarylheptanoid (Figure 3) [29].

(VI) Phenyl alcohols are also a relatively small class of phenols that contain a hydroxyl group that is linked to the aromatic ring by an alkyl group (C6–C2). The most important examples of this class are probably hydroxytyrosol and tyrosol. Esters of these alcohols with elenolic acid or its derivatives are an important subclass of phenols known as secoiridoids. While phenolic acids, phenolic alcohols and flavonoids occur in many fruits and vegetables, secoiridoids are present exclusively in plants belonging to the family *Oleaceae*, which includes *Olea europaea* L. Major examples of phenolic secoiridoids include oleuropein, oleacein and oleocanthal (Figure 3) [30].

## 4. Control of Oxidative Reactions by Antioxidants

**Figure 4:**
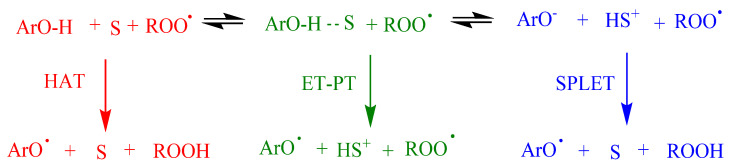
Proposed mechanisms between antioxidants (ArOH) and peroxyl radicals (ROO^•^) comprising the hydrogen atom transfer (HAT), electron transfer–proton transfer (ET-PT) and single-proton-loss electron-transfer (SPLET). S = Solvent. Adapted from reference [6].

The reaction of antioxidants, ArOH, with peroxyl, ROO^•^, radicals might take place through different mechanisms depending on structure of the antioxidant and the solvent: one-step hydrogen atom transfer (HAT), electron transfer-proton transfer (ET-PT), proton-coupled electron transfer (PCET) or sequential proton-loss electron transfer (SPLET), Figure 4. HAT and ET-PT often happen simultaneously, but the properties of the reactants, solvent polarity and ability to form hydrogen bonds are some of the factors that contribute to the precise mechanism.

Commonly, in aprotic solvents, ArOH reacts, most likely, through the HAT mechanism. However, the reaction rate may be lower if the solvent has the ability to form hydrogen bonds. In ionizing solvents such as water, methanol and ethanol, the phenolate anion can transfer an electron to the radical, and this mechanism is known as SPLET, where the reaction rate is highly affected by the acidity of the medium.

According to the mechanism in Figure 1, the rate of the inhibition reaction (r_inh_) between ROO^•^ radicals and ArOH (reaction 5, Figure 1) is given by Equation (2). A phenolic compound is an effective antioxidant if the inhibition rate is similar to or faster than the propagation rate (r_inh_ >> r_p_). In Equation (2), *k*_inh_ and *n* stand for the inhibition rate constant and the stoichiometric factor, that is, the stoichiometric factor of ArOH and number of peroxyl radicals trapped by ArOH [10].

(2) $$r_{inh}=nk_{inh}\left[ROO^{•}\right]\left[ArO-H\right]$$

The overall rate of the inhibition reaction depends, thus, on the values of the rate constant *k_inh_* of the pertinent reaction and the effective concentrations of reactants presented in each microenvironment of the multiphasic system. Table 2 includes some published inhibition rate constants for several antioxidants. The presence of different substituents in the chemical structure of antioxidants affects their reactivity, and this influence may be justified in terms of steric, resonance and inductive effects [31]. Therefore, the antioxidant efficiency can be affected by the number and position of hydroxyl groups linked to the aromatic ring and the nature, position and number of additional aromatic substituents, including alkyl chains with C = C double bonds, C=O groups and planar/non-planar structures.

**Figure 5:**
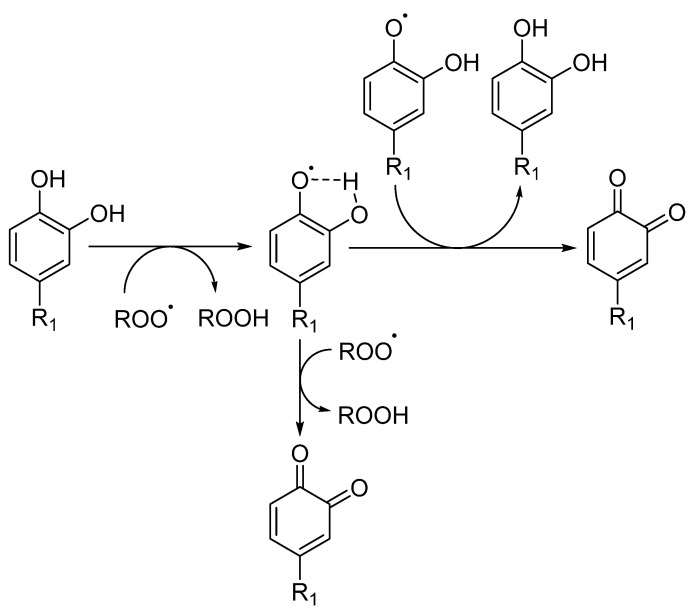
Radical scavenging activity of catechol derivatives, where R stands for different substituents.

The introduction of a second hydroxyl group into the 2 or 4 position of a phenol provides a higher reactivity than that observed for monohydroxy phenols due to the resonance-stabilized aryloxyl radical ArO^•^ (Figure 5) [10,31]. In catechols, the semiquinone radical stability is improved by intramolecular hydrogen bonding. Moreover, the presence and possible ionization of a carboxylic group in the structure of trihydroxy phenols, such as gallic acid, increases the hydrogen donation ability of phenolic acids, resulting in a higher inhibition rate constant than the one observed for pyrogallol (trihydroxy phenol without the presence of a carboxylic group) [10]. The introduction of oxygen, sulfur or nitrogen heterocyclic rings (e.g., tocopherol) linked to the phenolic ring also influences the rate of the antioxidant inhibition reaction, since the final rate will be given by the sum of the inductive factors and steric hindrance in the reaction with ROO^•^ radicals. The presence of ester substituents decreases the reactivity, because these functional group are effective hydrogen bond acceptors [32].

Determining the contribution of the effective concentrations of reactants to the overall inhibition rate is not an easy task in multiphasic systems where the oxidation of biomolecules may occur in different compartments, including the cell membrane separating the interior of the cell from the outside environment. In the absence of physical barriers, antioxidants partition between the three compartments at diffusion-controlled rates, and the global rate r_inh_ is described by Equation (3), where (ArO-H) and (ROO^•^) indicate the effective antioxidant and peroxyl radical concentrations, respectively, in moles per liter of the specific environment [10]. Here, the reader needs to consider that Equation (3) can only be defined under the assumption that the transport of reactants between different environments is not limited by physical barriers.

(3) $$\begin{array}{l} r_{\mathrm{inh}}=r_{\mathrm{inh}}{}_{\left(\mathrm{outside}\right)}+r_{\mathrm{inh}}{}_{\left(\mathrm{cell}\;\mathrm{membrane}\right)}+r_{\mathrm{inh}}{}_{\left(\mathrm{inside}\right)}=n\;k_{{\mathrm{inh}}_{\left(\mathrm{outside}\right)}}{\left({\mathrm{ROO}}^{•}\right)}_{\left(\mathrm{outside}\right)}{\left(\mathrm{ArO-H}\right)}_{\left(\mathrm{outside}\right)}+\\ n\;k_{{\mathrm{inh}}_{\left(\mathrm{membrane}\right)}}{\left({\mathrm{ROO}}^{•}\right)}_{\left(\mathrm{membrane}\right)}{\left(\text{ArO-H}\right)}_{\left(\mathrm{membrane}\right)}+n\;k_{{\mathrm{inh}}_{\left(\mathrm{inside}\right)}}{\left({\mathrm{ROO}}^{•}\right)}_{\left(\mathrm{inside}\right)}{\left(\text{ArO-H}\right)}_{\left(\mathrm{inside}\right)} \end{array}$$

Equation (3) raises the question of how the distribution and the effective concentrations of both antioxidants and free radicals in the different compartments can be estimated in order to know the inhibition rate.

Studies support that polyphenols can interact with the head groups of the membrane surface (adsorption) and with the bilayer interface (absorption), stabilized by forces including, mainly, hydrogen bonds and van der Waals interactions. For example, *trans*-stilbene resveratrol is mostly distributed in a deeper zone of the membrane, close to the lipid tails [34,35], although some studies indicate that it can be also located close to the polar headgroups.

Flavonoids can be distributed between the different regions of the membrane according to their chemical structure. However, as the number of hydroxyl groups increases, their hydrophobicity decreases, and their distribution change toward the lipid head groups [36]. It has been reported that galloyl flavan-3-ols distribute in the lipid tail region while the nongalloyl ones are located in the lipid–water interface [37]. Studies based on a molecular dynamics simulation also support that quercetin can be found in the lipid bilayer at different depths depending on if it is a glucuronide, a methylated or a sulfated metabolite obtained from conjugation reactions that take place in the liver during phase II of the metabolism of xenobiotics [36].

Likewise, it was observed that the membrane structure (fluidity or rigidity) can be modified, affecting their biological activity depending on the location of antioxidants and their local concentration [38]. In the literature, some studies showed that tea flavan-3-ols, kaempferol and quercetin can induce changes in the membrane structure, favoring the entrance of molecules of water into the membranes, while no significant changes were observed for the rosmarinic acid [37]. Using a series of alkyl caffeates, it was observed that caffeates have a much higher protective activity than caffeic acid against red blood cells’ (RBCs’) AAPH-induced oxidative hemolysis. However, the efficiency in this protection was heavily dependent on the length of the alkyl chain of the esters and on their concentration. At 2.5 and 5 µM, the more lipophilic caffeates (C8 and C16) showed a remarkable antioxidant activity, inhibiting hemolysis. Probably, their better location within the membrane leads to better antioxidative protection. However, at 50 µM, the more hydrophilic compounds (C1-C4) showed better activity against hemolysis than the more lipophilic ones (C8-C16). At this higher concentration, the better interaction of the more lipophilic compounds with the membrane seems to cause changes in RBC membrane fluidity, disturbing membrane integrity [39].

## 5. Importance of Partitioning of Antioxidants

Encapsulation, compartmentalization and accumulation of active ingredients in hosting agents and at the interfaces of surfactant-based colloidal systems (micelles, microemulsions, liposomes, emulsions, etc.) is of great interest to the food industry, material science and bioengineering [10]. For instance, the location of antioxidants at interfaces is central for numerous biological and technical processes. In biological systems, the transfer of antioxidants from the interior to the outermost (and vice versa) part of cells (or organelles) makes possible otherwise incompatible processes in the constrained space of the cells. In technology, approaches toward the entrapment of bioactives in 3-D phases are becoming quite common, and control of their delivery largely depends on how active components organize and distribute between the various domains of the system [10].

To build up quality and efficiency into a product and rationalization of formulation designs, a thorough understanding of the physicochemical properties of antioxidants is necessary. This requires a full characterization of the antioxidant prior to formulation design. For instance, the molecular characterization of the antioxidants by means of spectroscopic (NMR, EPR, IR, UV–vis, etc.) methods and the determination of key physicochemical properties are usually performed prior to any pre-formulation studies. The two key physicochemical parameters that are required to describe the distribution of the antioxidant between phases are the partition, *P*_W_^O^, and the ionization, K_a_, constants [10]. Both are intrinsic properties of the bioactive because their values depend on the chemical structure of the bioactive, which governs its interaction with the solvent.

The ionization constant, K_a_, is an intrinsic property of the molecule, whose value is closely related to its ability to donate hydrogen ions (H_3_O^+^) and describes the ionization behavior of the bioactive as a function of the acidity of the medium, determining the ratio between the ionized and non-ionized forms of the bioactive present in the solution, Equation (4). It is usually expressed as the negative logarithm of K_a_, pK_a_. When the pK_a_ equals the pH of the solution, the amount of ionized and neutral species is the same. The pK_a_ value is, thus, an important parameter in determining the ionization of the bioactive, and hence, its solubility in the aqueous phase.

(4) $$\mathrm{HA}\overset{K_{a}}{\;⇌}{\;H}^{+}{+A}^{-}\log\frac{\left[\mathrm{HA}\right]}{\left[A^{-}\right]}=pK_{a}-pH$$

It is worth noting that the neutral form is probably the only specie that diffuses through cell membranes but, as a drawback, it is the specie which has the lowest solubility in the aqueous phase [40,41]. Thus, understanding the distribution of ionized and neutral species is of maximum importance in predicting and quantifying their aqueous solubility, because ionized species are much more soluble than the neutral ones due to the ion-dipole intermolecular forces.

When solutes are added to partitioning systems (multiphasic systems), they distribute according to their solubility in the various phases, a phenomenon widely employed in a plethora of applications, including basic science and relevant economic areas such as medicine, pharmacy and food technology. Interest in investigating the partitioning of antioxidants comes, among others, from the fact that their solubilization in different environments results in different physicochemical interactions when compared to homogeneous systems. The distribution of solutes can be modified by changing the nature of the different phases (oil and surfactant types, etc.), composition and the experimental conditions (acidity, temperature, electrolyte concentration, etc.) [42].

The distribution of an antioxidant (and any other solute) between two immiscible phases is usually described in terms of the partition coefficient, *P*_W_^O^, Equation (5). This parameter, usually expressed in logarithmic form, log *P*_W_^O^, is determined as the ratio of the equilibrium concentrations of a neutral antioxidant in a model oil solvent (e.g., octanol, hexanol and edible oil) and an aqueous solvent [43]. *P*_W_^O^ values are measures of the relative hydrophobicity of a chemical substance, that is, how water loving (hydrophilic) or water fearing (hydrophobic) the antioxidant is. Antioxidants with high *P*_W_^O^ values are preferentially distributed to hydrophobic compartments such as oils and the lipid bilayers of cells, whereas chemicals with low *P*_W_^O^ values are preferentially localized in hydrophilic compartments such as the aqueous phase or blood serum.

(5) $$P_{W}^{O}=\frac{{\left[\mathrm{neutral}\right]}_{\mathrm{oil}}}{{\left[\mathrm{neutral}\right]}_{\mathrm{aqueous}}}$$

### 5.1. Properties of Antioxidants Affecting their Solubility in Bulk Solvents

The solubility process of an antioxidant (and, in general, that of any other solute) is usually rationalized in terms of three key energetic drivers. The first one is the necessary energy input to overcome the intermolecular interactions of the solute in its respective condensed state. The second is the energy input necessary to overcome solvent–solvent interactions and create a cavity in the solvent which accommodates the solute. This unfavorable energy input at this point is then countered with the energy release occurring upon the collapse of the solvent cavity around the solute and ensuing intermolecular interactions between solute and solvent.

Since antioxidant molecules contain different structures and functional groups, the collective contributions from each functional group make the macroscopic physicochemical properties of the antioxidant, which are a reflection of inter- or intramolecular interactions. For example, the stronger the attractions between antioxidant molecules are, the more difficult it is to separate them and, therefore, their solubility in a given solvent decreases [44,45].

Values of both intra- and inter-molecular forces are dictated by the values of intrinsic molecular properties, including the polarizability of the molecule, its lipophilicity and its ability to form hydrogen bonds with the solvent. The typical hydrogen bond is stronger than van der Waals forces but weaker than covalent or ionic bonds and can occur either intermolecularly or intramolecularly. Other factors such as surface areas, molar volumes and electronic factors may also be relevant in determining the solubility of a solute in a given solvent [45].

### 5.2. Properties of Solvents Affecting the Solubility of Solutes

For an antioxidant to be dissolved, both solute and solvent molecules must overcome their own intermolecular attraction forces and find their way between and around each other. This is accomplished best when the attractions between the molecules of both components are similar. The values of these attractive forces depend on the properties of the dielectric constant of the medium, which provides a rough measure of the solvent’s polarity. In general, polar solvents have higher dielectric constant values than nonpolar molecules. Solvents with a dielectric constant of less than 15 are generally considered nonpolar. The dielectric constants of some common solvents and cosolvents are listed in Table 3 [46].

As a rule of thumb, antioxidants solubilize better in solvents having similar polarities as that of the solute. Strongly polar antioxidants, such as phenolic acids, mainly dissolve in polar solvents such as water, whereas strongly apolar antioxidants such as tocopherols dissolve only in apolar solvents (edible oils, hexane, etc.)

### 5.3. Modifying the Distribution of Antioxidants in Partitioning Systems—A Brief Theoretical Background

When two or more immiscible solvents are present, solutes distribute thermodynamically between them according to their solubilities in each phase [10]. This is true assuming that saturation is not achieved in any of the solvents. Alterations in the molecular structure of the antioxidant and in that of the solvent can influence both solvent–solvent interactions and subsequent solvent–solute interactions and, therefore, their partitioning [45]. The extent of partitioning between regions or phases depends on the interaction of the antioxidants with the components of the various phases and, in binary oil–water systems where phases are at thermodynamic equilibrium, the transfer of solutes between phases can be described by the variation in the Gibbs free energy, ΔG^O→W^, associated to the transfer. ΔG^O→W^ is given by the difference in the chemical potentials, μ_AO_, of the molecule being transferred, ΔG^O→W^ = μ_AO_^O^ − μ_AO_^W^ (T, P constants) [6,10].

Thus, any effort to intentionally alter solubility will require a modification in the chemical potentials of either the solute solid state or the solute in solution, and this approach constitutes the basis for many of the ways to enhance the solubility of drugs in a given solvent. This can be achieved, for example, by creating alternative equilibria for the molecule, so that the chemical potential is modified through specific interactions, or by modifying its solvation. Some of the most common strategies are discussed in the next section.

### 5.4. Modifications to the Solvent Phase Affecting Partitioning (Table 4)

- Cosolvents: The addition of cosolvents has the ability to alter the dielectric constant of a given solvent, affecting, therefore, the energy required to overcome hydrogen bonding forces in aqueous media and reducing the amount of energy required to create cavities in the mixture to accommodate the solute. Moreover, these changes in the solvent properties can greatly alter the degree of solvation of the solute, eventually affecting the partitioning of the molecule.
- Hydrotropes: The addition of hydrotropes is commonly employed to increase the water solubility of poorly soluble drugs, and, in many cases, the water solubility is increased by several orders of magnitude [47]. Examples of hydrotropic agents include urea, caffeine, tryptophan, etc.
- Micellar systems: Surfactants are amphiphilic molecules having both hydrophobic and hydrophilic portions that, when added to solution, associate spontaneously to form spherical aggregates—micelles—once the surfactant concentration reaches a critical value, called the critical micelle concentration. The hydrophobic portions of surfactants are chains with 8–22 carbon atoms. Common hydrophilic groups of ionic surfactants are carboxylate (–COO^−^), sulfate (–OSO_3_^−^), sulfonate (SO_3_^−^), carboxybetaine (–NR_2_CH_2_COO^−^), sulfobetaine (–N(CH_3_)_2_C_3_H_6_SO_3_^−^) and quaternary ammonium (–R_4_N^+^). The hydrophilic group of nonionic surfactants is usually a polyoxyethylene group, but glyceryl groups or sorbitol groups are also common, and their use depends on the particular application. Surfactants commonly used in food and pharmaceutical products include, but are not limited to, non-ionic surfactants such as polysorbates, polyoxyethylenes, triton X, Cremophor EL and Chremopor RH60.

Micellar aggregates contain a hydrophobic core (e.g., in O/W systems) that allows solubilization of hydrophobic molecules that otherwise cannot be dissolved in the aqueous matrix. The partitioning effect is usually quantified in terms of the association constant of the molecule to the aggregate, Equation (6), where SW stands for the solute in water, SM the solute in the micelle, Dn is the micellized surfactant (Dn = [Surf]total – CMC) and KS stands for the association constant of the molecule to the micellar aggregate.

(6) $$S_{W}{+D}_{n}\overset{K_{S}}{⇌}{\;S}_{M}\Rightarrow K_{S}=\frac{{\left[S\right]}_{M}}{{\left[S\right]}_{W}D_{n}}$$

- Liposomes: Liposomes are closed spherical vesicles composed of one or more bilayers of amphipathic lipid molecules enclosing one or more aqueous core compartments [48]. Hydrophobic antioxidants can be solubilized by liposomes, becoming an integral part of the lipid bilayer. Water-soluble drugs reside within the aqueous inner core and are released as the liposome erodes in vivo or by leakage. A typical liposome formulation contains water with phospholipid at ~5–20 mg/mL, an isotonicifier and a pH 5–8 buffer. The phospholipids normally used include phosphatidylcholine, phosphatidic acid, phosphatidylglycerol and saturated lipids such as l-a-dimyristoylphosphatidylcholine (DMPC), dipalmitoyl phosphatidylcholine (DPPC), dipalmitoyl phosphatidic acid (DPPA) and l-a-dimyristoylphosphatidylglycerol (DMPG).
- Microemulsions: Microemulsions are a thermodynamically stable isotropically clear dispersion composed of a polar solvent (usually water), an oil, a surfactant and a cosurfactant. Microemulsions offer many advantages compared to macroemulsions; they have smaller particles (often <100 nm), require less energy to be produced and have higher physical stability, due to the high levels of emulsifiers used in their preparation [49]. Microemulsions generally have very low interfacial tension at the water–oil interface and form a highly fluid interfacial surfactant film. Due to the numerous small droplets, the surface area-to-volume ratio of microemulsions is very high.

**Figure 6:**
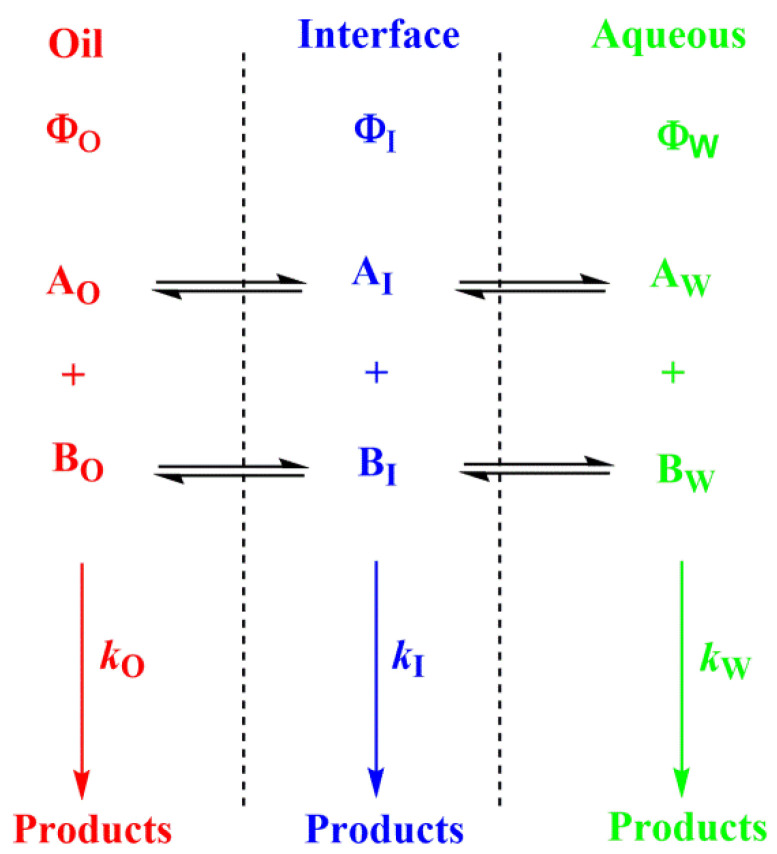
Distribution of reactants between the different regions of a multiphasic system, such as a microemulsion. Φ and *k* stand for the volume fraction in each particular region and for the second order rate constant for the reaction between A and B in each region, respectively (O–oil, I—interfacial and W—aqueous regions).

The presence of surfactant and cosurfactant could make microemulsion supersolvents for drugs relatively insoluble in both aqueous and hydrophobic solvents [50]. Sometimes mixed oils and/or mixed surfactants are employed in the preparation of microemulsions, offering some advantages over pure single component materials [50]. Quantifying the distribution of solutes in microemulsions is difficult. Nevertheless, once the interfacial polarity and microstructure are known, along with how compounds may interact with the different phase solvents, qualitative predictions can be made, Figure 6.

- Emulsions: Emulsions are, in contrast with microemulsions, thermodynamically unstable mixtures of oil and water in which one liquid is dispersed as small spherical droplets in the other one. Since the molecules of the liquids are in direct contact with each other, they tend to separate and, on standing, emulsions phase separate through different processes to minimize the contact between them. Emulsions, however, can be stabilized kinetically by adding a surfactant or emulsifier. The added surfactants are arranged on the interface between the oil and water phases so that the hydrophilic part of the surfactant is located in the polar phase; meanwhile, the hydrophobic portion tends to be located in the oily phase, so that surfactants create a film surrounding the surface of the droplets, resulting in a decrease in the interfacial tension and ensuring kinetic stability for some time [49,51].

When antioxidants are added to emulsions, they distribute, as in microemulsions, thermodynamically between the oil, interfacial and aqueous regions, so that two partition constants are necessary to describe its distribution, that between the oil–interfacial, POI (Equation (7)) and that between the aqueous–interfacial, PWI (Equation (8)) regions, Figure 6.

(7) $$P_{O}^{I}=\frac{\left(AO_{I}\right)}{\left(AO_{0}\right)}$$

(8) $$P_{W}^{I}=\frac{\left(AO_{I}\right)}{\left(AO_{W}\right)}$$

**Figure 7:**
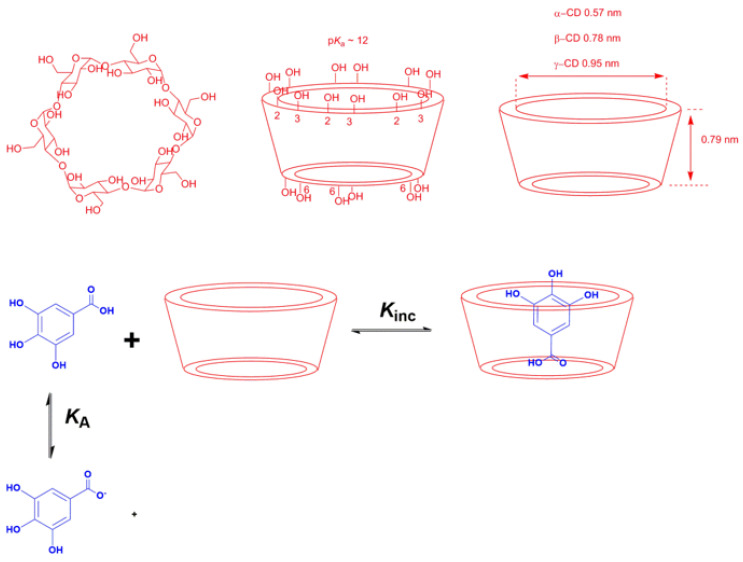
Chemical structures of the natural CDs showing the spatial conformation, the location of primary and secondary –OH groups and the size of their cavities, and an illustrative representation of an inclusion complex between a gallic acid derivative and cyclodextrins (CDs) [53].

- Complexation: The main strategy of complexation is to reduce the exposure of the molecule’s hydrophobic region to water. This can be achieved, for instance, by adding water-soluble agents capable of hosting water-insoluble molecules. Crown-ethers and curcubiturils contain hydrophobic cavities capable of hosting hydrophobic molecules, but probably the most widely employed hosting agents are the cyclodextrins (CDs) because of their high water-solubility and in vivo safety margin, Figure 7 [52].

The inclusion of antioxidants in a CD cavity can be rationalized in terms of the substitution of the water molecules included in the cavity by the somewhat hydrophobic antioxidant. The host–guest complexes are non-covalent because no covalent bonds are formed or broken during their formation, being the driving forces for the inclusion process in hydrophobic interactions, hydrogen bonds and Van der Waals interactions [54,55]. Antioxidants, however, may readily dissociate in aqueous solution, and the equilibrium between free antioxidant molecules and hosted antioxidants is rapidly established.

The formation of CD complexes is, thus, a dynamic equilibrium process [56] that can be described by Equation (9), where *m* and *n* are the stoichiometric coefficients, CD is the cyclodextrin, AO is the guest molecule (in this work, an antioxidant) and AO-CD is the inclusion complex, respectively. The most typical stoichiometry is 1:1, but 1:2, 2:1 or even 1:3 complexes have been reported [57]. For a 1:1 complex, the equilibrium constant K_C_ for the formation of the inclusion complex is given by Equation (10).

(9) $$nAO+mCD\overset{k_{F}}{\underset{k_{D}}{⇄}}AO_{n}-CD_{m}$$

(10) $$K_{C}=\frac{\left[AO_{n}-CD_{m}\right]}{{\left[AO\right]}^{n}{\left[CD\right]}^{m}}$$

- Combined strategies: Decisions regarding the optimal strategy to modify the partitioning of antioxidants often lie in modifying, in some way, the intrinsic solubility of the antioxidants in a given phase. Thus, combinations of methods, such as variations in the acidity or temperature of the solution together with the use of surfactant-based systems and cyclodextrins, have been widely used. Technologies such as cosolvency and pH modifications are also commonly employed to shift equilibria. Increasing the salt concentration affects partitioning due to the ion-pair association, salting-in or -out effects and because the composition of phases changes.

### 5.5. Modeling the pH-Dependent Partition Constants in Binary and Multiphasic Systems

As shown by Equation (4), the fraction of neutral and ionized species is governed by the acidity of the aqueous phase and the pK_a_ of the antioxidant. Therefore, the actual *P*_W_^O^ values need to be considered as “apparent” values, only valid under given conditions because they depend on the chemical nature of the antioxidant and on the particular experimental conditions employed. It is, thus, necessary to predict how the partition constant of a given antioxidant changes upon changing the acidity of the aqueous solution [40,41].

**Figure 8:**
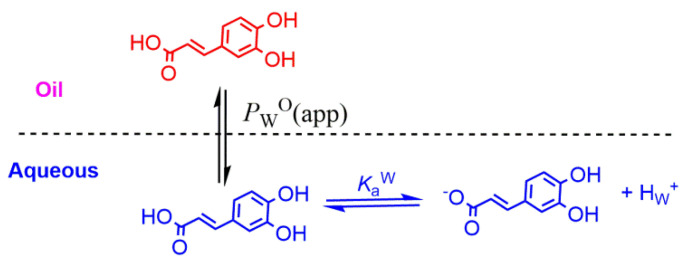
Partitioning of a model phenolic acid antioxidant (caffeic acid) between the oil (O) and water (W) phases of binary mixtures.

When neutral antioxidants distribute between two immiscible phases (e.g., oil and water) at equilibrium, their partition constant *P*_W_^O^ between those phases is defined by Equation (5). If the antioxidant has an ionizable group (e.g., a phenolic acid such as caffeic acid (CA)), a fraction of it may be ionized and both the neutral and ionized species are in equilibrium between the oil and water phases, Figure 8. Reported ionization constants of acids in oils (O) are usually much lower (5–6 orders of magnitude) than those in aqueous solution (i.e., pK_a_(O) >>> pK_a_(W)) and, therefore, only the neutral form of the antioxidant partitions into the oil phase. Thus, and for the sake of simplicity, it is frequently assumed that the ionization of the weak acids in the oil phase is negligible, Figure 8, from where the apparent (measured) partition coefficient, defined by Equation (11), can be derived for any phenolic antioxidant AH.

(11) $$P_{W}^{O}\left(\mathrm{app}\right)=\frac{{[\mathrm{AH}}_{\mathrm{oil}}]}{{[\mathrm{AH}}_{\mathrm{water}}]+[A_{W}^{-}]}$$

Bearing in mind the definition of the dissociation constant of the acid, *K*_a_, Equation (12) can be easily derived. This equation sets the relationships between the apparent partition constant *P*_W_^O^ (app) and the acidity (pH) of the aqueous phase.

(12) $$P_{W}^{O}\left(\mathrm{app}\right)=\frac{P_{W}^{O}}{1+\frac{K_{a}}{{[H}^{+}]}}$$

(13) $$\frac{{(\mathrm{AH}}_{W})}{{[\mathrm{AH}}_{T}]}=\frac{1}{\Phi_{W}+P_{W}^{O}\Phi_{O}+\frac{K_{a}}{{[H}^{+}]}}$$

The local concentration of an antioxidant in the water phase can be obtained by bearing in mind the corresponding mass balance for the concentration of the acid in terms of the total volume of the system, and Equation (13) can be derived, where AH stands for the antioxidant, brackets [ ] means concentration in moles per liter of total volume, parenthesis ( ) stand for the concentration in moles per liter of phase volume and Φ_O_ = V_oil_/(V_o_ + V_w_) and Φ_W_ = V_w_/(V_o_ + V_w_) stand for the volume fraction of the oil phase and aqueous phase, respectively [40,41].

**Figure 9:**
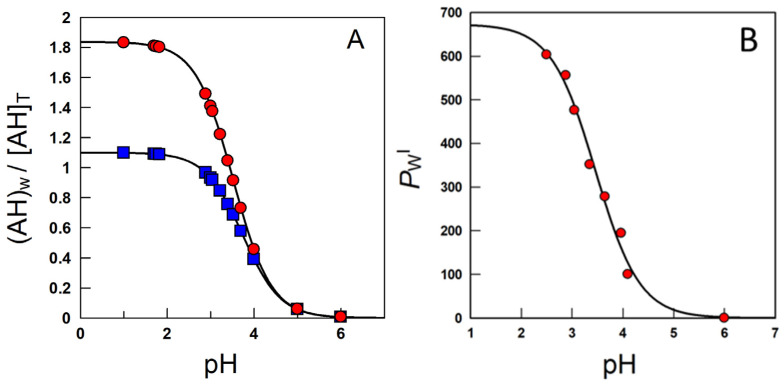
(**A**) Variation with pH of the ratio between the local concentration of the caffeic acid (here represented as AH) in the aqueous region and the total or stoichiometric concentration obtained in corn oil–water systems, T = 25 °C, at two o:w ratios (■—1:9 and ●—1:1, *v*/*v*). (**B**) Effects of acidity on the partition constant *P*_W_^I^ of caffeic acid in emulsions [58].

Equation (13) is a modification of the Henderson–Hasselbach equation and gives the concentration of the neutral weak acid in a two-phase system relative to the total (stoichiometric) concentration as a function of the acid concentration, the volume fractions of each phase, the partition constant of the neutral molecule and the ionization constant. Figure 9A simulates the variation in the ratio between the local concentration of the AO in the aqueous region and the total or stoichiometric concentration at two o:w ratios.

Similar equations have been developed to quantify the variations in the local concentrations of solutes in association colloids and cyclodextrins, which can be considered as two-site model systems, Figure 7 [57].

**Figure 10:**
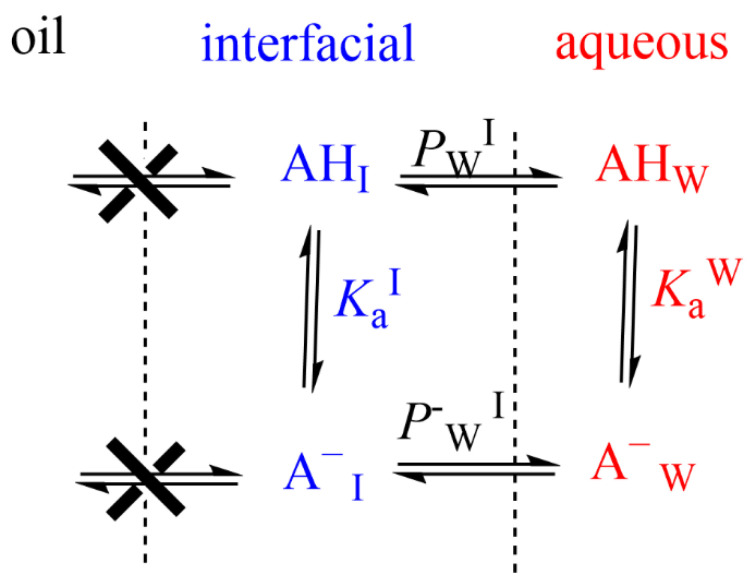
An illustrative representation of the distribution of an oil insoluble carboxylic antioxidant (AH) between different regions of an emulsified system. *P*_W_^I^ and *P*^−^_W_^I^ stand for the partition constants for the neutral and ionized species of the carboxylic antioxidant.

In emulsions, for oil insoluble AOs such as gallic or caffeic acids, only the variation in the partition constant between the aqueous and interfacial regions needs to be considered [40]. The ionization constant of the carboxylic group of these phenolic acids in the aqueous region, *K*a^w^, is assumed to be similar to that in the interfacial region, *K*a^I^, Figure 10, and the apparent distribution constant of the protonated AO can be derived in terms of the partition and ionization constants, Equation (14), where *P*_W_^I^, and *P*^−^_W_^I^ stand for the partition constants for the neutral and ionized species of the carboxylic antioxidant, respectively. Details can be found elsewhere [40,58].

(14) $$P_{W}^{I}\left(\mathrm{app}\right)=\frac{P_{W}^{I}+\frac{{{P^{-}}_{W}}^{I}K_{a}}{{[H}^{+}]}}{1+\frac{K_{a}}{{[H}^{+}]}}$$

At high acidities, *K*_a_/[H^+^] <<1, the (neutral) antioxidant is basically distributed between the aqueous and interfacial regions, so that *P*_W_^I^ (app) ≈ *P*_W_^I^. Upon lowering the acidity, the concentration of the ionized form increases and, eventually, *K*_a_/[H^+^] >> 1 and *P*_W_^I^(app) ≈ *P*_W_^I^ [H^+^]/*K*_a_ + *P*^−^_W_^I^ ≈ *P*^−^_W_^I^. *P*_W_^I^(app) values decrease upon increasing pH because *P*_W_^I^ should be >>> *P*^−^_W_^I^. Figure 9B is illustrative of the variations in *P*_W_^I^ with the pH for caffeic acid, as a representative phenolic acid [40].

## 6. Diffusion and Transport of Reactive Species and Antioxidants

**Figure 11:**
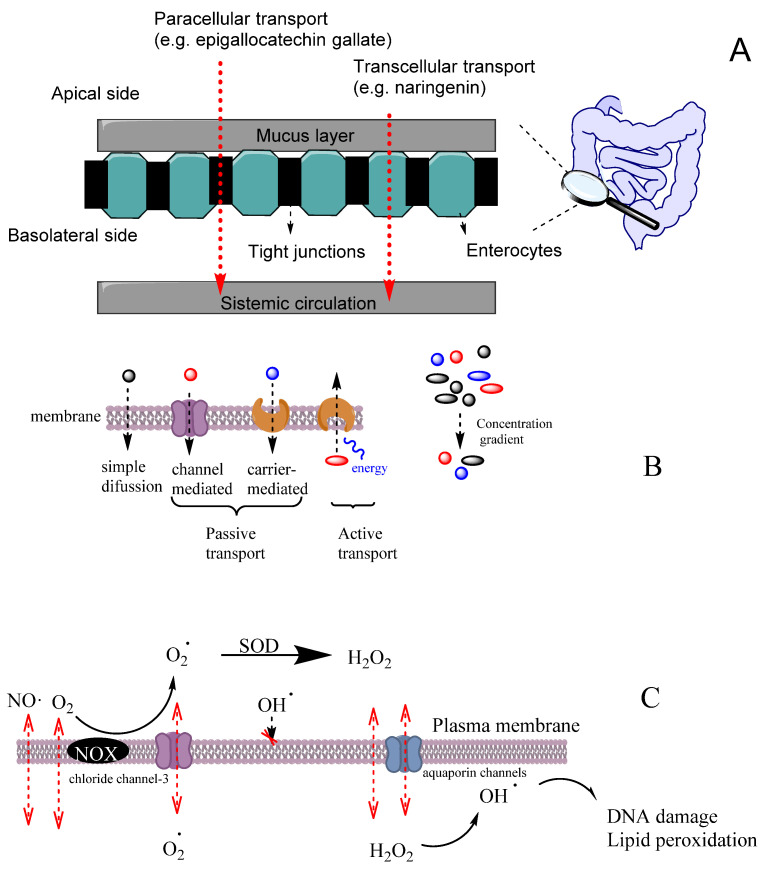
(**A**) Different transport pathways across the intestinal cell epithelium. Adapted from [69], Copyright (2022), with permission from Elsevier. (**B**) General types of membrane transport. (**C**) Permeability of lipid membranes to different reactive species [60,61,62]. Adapted from reference [60], Copyright (2022), with permission from Elsevier.

An efficient antioxidant is an antioxidant that is able to distribute in compartmentalized systems with the ability to diffuse readily to the site where the oxidative reactions are taking place so that the rate of the inhibition reaction is higher than that for the production of radicals. Enterocytes form a barrier to antioxidants that offers only a very limited passage of molecules in both directions. Moreover, cell–cell adhesion is ensured by tight junctions, desmosomes and adherent junctions. Therefore, molecules can cross the enterocytes barrier by a transcellular (crossing the cell membranes) or by a paracellular (between cells) route depending on their physicochemical properties and their capacity to interact with the membrane, Figure 11A [38].

Cell membranes are selective barriers that compartmentalize different processes inside and outside of cells. Cell membranes are usually described by the fluid mosaic model [59] as a fluid lipid bilayer (composed of different types of lipids including phospholipids, glycolipids and cholesterol) containing different types of proteins and other organic molecules (e.g., tocopherol). Lipid and protein components of membranes change according to the type of the cell and their location. Cell membranes may have a high concentration of transmembrane proteins that change lipid packing, restringing lateral molecular membrane diffusion. Two main zones can be differenced in the membrane that can act as potential barriers to diffusion, depending on the physicochemical properties of the molecule. The first zone, the polar headgroups region, characterized with a high ability to form H bonds, is where water molecules interact with the lipid bilayer. The second zone, containing mostly fatty acid acyl chains, is characterized by a high hydrophobicity. For most hydrophobic molecules, the first zone may present a small thermodynamic barrier to its diffusion, whereas, in the case of polar molecules, the barrier to its diffusion may occur in the zone of the alkyl chains of the acyl groups.

The transport of molecules across cell membranes can be achieved by active or passive transport, Figure 11B [60]. Passive transport can be achieved by simple diffusion, facilitated diffusion involving protein channels or osmosis (in the case of water molecules). In this case, the movement of a molecule across membranes does not require energy, and the diffusion process finishes when the concentration of the molecule in both sides of the membrane attains equilibrium, driven by the potential energy difference of a concentration gradient. Active transport involves the movement of molecules through protein channels against their concentration gradient and needs energy, commonly in the form of ATP.

The ability of a molecule to cross the membrane is defined by the permeability coefficient (Equation (15)), where δ is the thickness of the membrane (approx. 4 nm), Dm is the diffusion coefficient of the molecule in the membrane and *P* is the partition coefficient between the membrane and water [61]. Equation (15) considers that both molecule solubility and diffusion do not change at different depths in the membrane. The passive diffusion of a molecule can be predicted following the basic models of linear diffusion, such as Fick’s first law, applied to the concentration gradient inside the membrane (where J is the flux in molcm^2^s^−1^, C°_m_ and C^δ^_m_ are the concentrations at the membrane boundaries (mol/cm^3^), δ is the thickness (cm) of the membrane, Equation (16)) [62].

(15) $$P_{m}=\frac{D_{m}P}{\delta }$$

(16) $$J=D_{m}\frac{dD_{m}}{dx}=D_{m}\frac{\left[C_{m}^{0}-C_{m}^{\delta }\right]}{\delta }$$

Cells employ several mechanisms to transport substrates depending on their physicochemical properties. Different signaling molecules, bioactive and waste products are routinely transported (received and delivered) across cell membranes. The efficient delivery of bioactives and other products to the right locations within a cell is crucial to normal cellular function and to maintain homeostasis. Many physiological processes depend on selective exchange of bioactives between the cell and its exterior. However, the exact mechanism by which compounds cross cell membranes and reach the target locations is still not fully understood [63].

Oxygen and some reactive species such as NO^•^ or NO_2_^•^ have quite high Pm values and, therefore, they can easily cross membranes by simple diffusion. These species have an even higher Pm than those of hydrogen peroxide or superoxide (Table 5). However, hydrogen peroxide and superoxide can have their permeation rate increased because H_2_O_2_ may use aquapores and O_2_^−^ may use anion channels to cross membranes, Figure 11C [61]. In contrast, the diffusion of hydroxyl radical (OH^•^) is quite restricted due to its very high reactivity, being scavenged by almost any molecule or by the membrane polar head group before it can diffuse across the membrane. [61].

Another important mechanism of transport is the vesicular one [62]. Vesicles are involved in the transference of large molecules or bulky materials taken up at the cell surface (endocytosis) and are responsible for molecular traffic between a variety of specific membrane-enclosed compartments. Endocytosis is a general term for the various types of active transport that move particles into a cell by enclosing them in a vesicle made out of plasma membrane [70].

There are variations in endocytosis, but all of them follow the same basic process. First, the plasma membrane of the cell invaginates, forming a pocket around the target cargo [70]. The pocket then pinches off with the help of specialized proteins, leaving the cargo trapped in a newly created vesicle. The delivery of the transport vesicle at the target membrane usually involves the correct recognition of the target membrane and its fuse with the membrane (which can be a cell or an organelle membrane), thereby delivering its content. This is, for example, how low-density lipoproteins are delivered to cells. However, some vesicles, such as mixed micelles, obtained during lipid digestion containing phospholipids, cholesterol, fatty acids and other dietary molecules such as tocopherol and carotenoids are able to deliver their content by fusing with the enterocyte membrane without the need for a specific recognition [71].

Different strategies have been employed to understand the potential mechanisms by which antioxidants and their metabolites cross membranes, ranging from in silico, in vitro and in vivo models [63]. For some compounds, including vanillic acid, 4-hydroxybenzoic acid, caffeic acid, protocatechuic acid and ferulic acid, the in silico estimate of brain permeability was confirmed by in vivo results. However, this validation was not reflected for other compounds [63]. For example, gallic acid was found at significant concentrations in the rat brain [72], although the SWISS ADME model predicts that it should not be able to cross the hematoencephalic barrier by passive diffusion. These results can be explained by the fact that the in silico studies only considered permeation by passive diffusion and did not consider the possibility of other types of transport.

Interesting studies are being addressed in order to promote the antioxidants’ membrane permeability and stability by developing different antioxidant delivery strategies. For example, sinapic acid conjugated with a polymer has been encapsulated in bovine serum albumin-based nanoparticles and tested as a potential blood–brain–membrane-permeable form for its delivery into the brain [73]. The efficient delivery of bioactives to select locations within a cell is of key importance to normal cellular function.

## 7. Bioactivity of Antioxidants: Mechanism(s) of Action

Although the bioactivity of antioxidants has been described in numerous studies, beneficial health effects appear to be more related to a diet rich in these compounds than to taking supplements containing individual compounds. In some cases, situations have even been found in which the ingestion of certain pure antioxidants has been shown to have adverse effects on specific populations [74]. This difference in the results obtained may be due to interactions of antioxidants with compounds existing in the food matrix or to a greater stability of AOs to the gastrointestinal conditions when ingested in the food matrix [75]. Thus, the location of antioxidants within cells and the richness of the food in other dietary antioxidants may contribute to greater bioactivity [75]. It also should be taken into account that many physiological processes depend on the formation of free radicals, and these can be inhibited due to an exaggerated supplementation of antioxidants that is usually avoided when antioxidants are taken from natural diet sources. For example, physical exercise is positively correlated with longevity and decrease in insulin resistance in patients with type 2 diabetes mellitus. However, physical exercise increases the production of reactive oxygen species in mitochondria that, at first glance, one should think to be harmful. It was found that supplementation with vitamin C (1000 mg/day) and vitamin E (400 IU/day), two of the body’s main antioxidants, inhibits the benefits obtained by exercise. Thus, only the group that exercised without vitamin supplementation showed greater insulin sensitivity and higher levels of plasma adiponectin.

The mechanisms by which phenolic compounds can exert their beneficial effects on human health can be various [76]. The most obvious mechanism of action in the prevention of cellular oxidative damage should be a consequence of their antiradical properties and their ability to modulate biological oxidative stress, preventing damage of lipids, proteins and DNA, since they show a high direct radical scavenging capacity against numerous nitrogen and oxygen reactive species (e.g., hydroxyl and peroxyl radicals). From the thermodynamic point of view, their reactions with superoxide anions, singlet oxygen and lipid peroxyl radicals are favorable [77]. As already mentioned, these compounds may also exert their antioxidant activity indirectly, either through the capacity of recycling endogenous antioxidants (e.g., glutathione, urate, vitamin E and vitamin C) or through the capacity of some of these compounds, such as catechins and quercetin, to chelate metallic ions (Fe and Cu, important initiators and catalysts of oxidation reactions) [78].

Given the importance of reactive oxygen species in biological systems, the reactivity of antioxidants with RONs has been widely studied. Generally, the enzymatic system (hypo)xanthine-xanthine oxidase (X/XO) and non-enzymatic sources (potassium superoxide) are used as sources of the superoxide anion in the evaluation of antiradical activity. Many natural antioxidants (e.g., quercetin, myricetin, rutin, etc.) are able to scavenge superoxide anions generated from the X/XO system [79], but their scavenging capacity depends on the particular chemical structure. In the case of flavonoids using the X/XO system in the assessment of their antiradical capacity, the antioxidant activity can occur either directly by direct scavenging of radicals or by inhibiting the activities of free radical-generating enzymes such as xanthine oxidase and nitric-oxide synthase. The structure–activity relationships of flavonoids as inhibitors of xanthine oxidase and as scavengers of the superoxide radical [80] showed that the presence of hydroxyl groups at carbons 5 and 7 and the double bond between carbons 2 and 3 is essential for a high inhibitory activity on xanthine oxidase. However, for high superoxide scavenging activity, the presence of an ortho-dihydroxy substitution in the B-ring seems to be the most important feature for antioxidant activity [81]. Flavonoids may have intramolecular hydrogen bonds that decrease the antioxidant activity of hydroxyl groups when acting as hydrogen bond donors (5− OH, 3− OH and 3′− OH, Figure 3) while enhancing the antioxidant activity of hydroxyl groups, acting as hydrogen bond acceptors (4′− OH, Figure 3). In water, several flavonoids may suffer consecutive proton loss reactions from all of the OH groups with the formation of polyanions. These proton loss reactions usually begin at the 7−OH group. The second proton loss reaction usually involves the -OH groups on the B ring due to the better delocalization of the negative charge over the entire molecule [81]. Therefore, these polyanions are able to scavenge three free radicals via three consecutive ET reactions [81]. The free radical scavenging activity can also be promoted by double bonds in the phenolic ring side chains and by the glycosylation. Glycosylation of cyanidin to cyanidin-3-glucoside and malvidin to malvidin-3-glucoside, for example, had similar effects of enhancing antioxidant activity [82].

The hydroxyl radical is more reactive than the superoxide anion, thus producing greater tissue damage. Most antioxidants are highly reactive towards the hydroxyl radical, with scavenging rates 100–300 times higher than that of mannitol, a typical hydroxyl radical scavenger. The rate constants of quercetin and kaempferol with the hydroxyl radical are 4.3 × 10^9^ M^−1^ s^−1^ and 4.6 × 10^9^ M^−1^ s^−1^, respectively, which are about four orders of magnitude higher than those with the superoxide anion [83]. Some antioxidants can also inhibit the consumption of O_2_ (respiratory burst) by human neutrophils due to their inhibitory action on NADPH oxidase, the enzyme responsible for reducing O_2_ to the superoxide anion. Consequently, the production of superoxide anions and hydrogen peroxide decreases significantly in the presence of antioxidants. Antioxidants can also significantly inhibit the activity of myeloperoxidase contained in neutrophils, which is responsible for producing strong oxidants such as HOCl by the oxidation of chloride (Cl^−^) by H_2_O_2_. Moreover, nitric oxide (NO^•^) has an important role in neurotransmitter and second messenger function, but it becomes harmful once its concentration increases beyond a certain concentration because it reacts with the superoxide anion generating peroxynitrite (ONOO-), which is a very reactive oxidant. The ONOO- scavenging activity of some polyphenols, such as flavonoids, was found to be 10 times higher than that of ebselen, an efficient peroxynitrite scavenger [81]. Several flavonoids, including quercetin, can reduce ischemia–reperfusion injury by inhibition of inducible NOS (iNOS) activity. In addition, some antioxidants have been shown to inhibit cyclooxygenases, lipoxygenase, microsomal monooxygenase and NADH oxidase [76].

Antioxidants may also act as hydrogen donors to the α-tocopherol radical, diminishing LDL oxidation through interactions with the α-tocopheryl radical. Some flavonoids (e.g., quercetin, epigallocatechin gallate and naringin) and phenolic secoiridoids can exhibit strong inhibitory activity against LDL oxidation in vitro [84].

**Figure 12:**
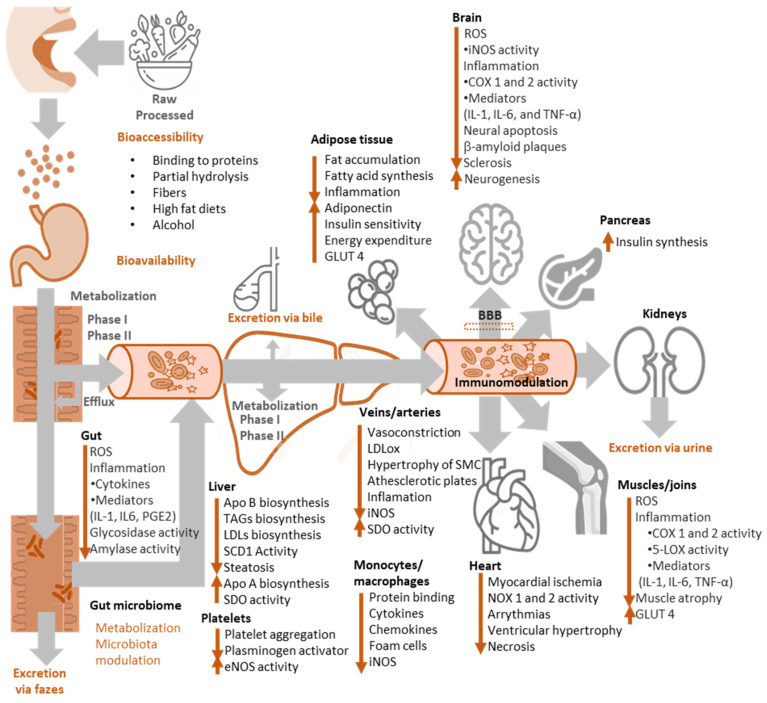
Fate of antioxidants in the body and main bioactivities attributed to them.

However, the biological relevance of these mechanisms of action has been questioned because of the low concentrations of exogenous antioxidants compared to those of endogenous chelating and antioxidant compounds (glutathione, urate, vitamin C, ferritin, etc.) [81]. Moreover, the bioactivity of phenolic compounds in vivo greatly depends on their metabolism (Figure 12), but phenol-conjugated metabolites have frequently shown a much lower radical scavenging capacity than the parent compounds and, unless some intracellular conjugation occurs, they are quite inactive as antioxidants [85]. Nevertheless, and considering that dietary phenols are consumed throughout the human life, their accumulation can result in antioxidants with low radical scavenging capacity and may contribute to the overall antioxidant protective effects [86]. Moreover, the possible accumulation in a particular tissue [87], their link to cell proteins and lipoproteins [88] and the possible additive or synergetic effects between all dietary phenols [89] are not well known and are probably underestimated in in vivo studies. In fact, food and its components are ingested throughout a lifetime, during which even modest antioxidant effects may become noteworthy.

Studies carried out with the consumption of fruits and vegetables also raise other questions, since components of these foods, other than polyphenols, can increase the antioxidant capacity of plasma. This is the case wherein fructose, found in fruits and some vegetables, increases the plasma antioxidant capacity by increasing plasma urate levels through the metabolism of fructose mediated by frutokinase [90]. Thus, studies carried out with foods rich in phenols should consider the effects of non-phenolic components in the food matrix, such as fructose, sucrose and sorbitol content.

Most studies carried out in humans show that the consumption of foods rich in polyphenols exerts beneficial physiological effects on health through an improvement in endothelial function, platelet function, arterial elasticity, blood pressure, insulin sensitivity and the blood lipid profile. Given the low concentration of these compounds obtained by diet, the mechanism of action of polyphenols should require interactions of high molecular specificity with cellular structures in order for a certain biological effect to occur. For example, a phenol–protein interaction can dramatically alter a plasma membrane protein which, in turn, can significantly alter various biological functions, even at the low concentrations of polyphenols achievable through the diet. They may also indirectly exert their antioxidant activity by stimulating endogenous antioxidant defense systems (e.g., glutathione peroxidase and superoxide dismutase) and/or inhibiting enzymes that generate large amounts of ROS, such as xanthine oxidase and NAD(P)H oxidase. Another mechanism associated with some polyphenols is their ability to inhibit the absorption of oxidized products, such as lipid hydroxyperoxides [91]. Nevertheless, recent studies have reported that most of the physiological effects of polyphenols are being mediated by their ability to modulate gene and protein expression [92].

The majority of the effects attributed to phenolic compounds are, therefore, linked to multiple mechanisms involving signaling pathways and the modulation of molecular events associated with differentiation, proliferation, cell survival, angiogenesis, detoxification and antioxidant enzymes, hormonal activities and immune responses. Despite their promising role in disease prevention and treatment, polyphenols often have poor bioavailability, representing an important limit to their use. However, the bioavailability of these compounds can be improved by several strategies, namely, their administration in combination with other phytochemicals and drugs or in polyphenol loaded, nanotechnology-based delivery systems.

## 8. Bioavailability of Antioxidants

The positive effects of antioxidants on health depend considerably on their bioaccessibility/bioavailability. Bioaccessibility is defined as the amount of ingested antioxidant that is available for absorption in the intestine after digestion, while bioavailability refers to the fraction of ingested antioxidant that reaches the systemic circulation and the specific sites where it can exert its biological action. Most antioxidants have apparently low bioaccessibility/bioavailability, due to, among other factors, the interaction with the food matrix (proteins, fiber, fats), food processing, availability and activity of individual digestive enzymes and mucines, metabolism in the intestine and liver and, often, they suffer from the action of microbiota. Once ingested, polyphenols can suffer mechanical disruption by chewing and crushing in the mouth, which is a critical step in the liberation of the polyphenol from the food matrix and interaction with saliva components.

The bioavailability of phenolic antioxidants heavily depends on their physicochemical characteristics, e.g., the molecular weight, structure and glycosylation degree, meaning that the metabolic path followed by each molecule can be quite different from one to another, even within the same class of phenols. Therefore, the most abundant antioxidants in foods may not be the most bioavailable or those that have the greatest bioactivity. Small molecules, such as phenolic alcohols, phenolic acids and flavonoids, are generally easily absorbed in a dose-dependent manner. However, only their metabolites are detected in plasma, even though they may be present in plasma as parental compounds [93]. On the other hand, polymeric polyphenols, such as proanthocyanidins, are poorly absorbed [94]. After ingestion, antioxidants must achieve the circulatory system. In the stomach, due to its high acidity levels, non-glycoside, liposoluble phenols can be absorbed by passive diffusion [95]. However, the relatively low time of residence in the stomach and the presence of mucins lining the gastric mucosa prevents most of their absorption due to interactions with glycoproteins. Some phenols can also be trapped in the fiber. The great variety of polyphenols and complexes they may form with food and extracellular components increases the complexity of their availability. Moreover, as most phenols in nature are in the form of glycosides, they usually have poor liposolubility, decreasing their transport through membranes. Therefore, the attached sugar moiety needs to be hydrolyzed, releasing the more lipophilic aglycone, before absorption [95]. Polymeric polyphenols and phenols containing glycosidic and ester bonds may suffer a partial hydrolysis in the stomach. For example, procyanidin can be partially hydrolyzed to epicatechin, oleuropein can be partially hydrolyzed to its aglycone and oleacein can be partially hydrolyzed to hydroxytyrosol in the gastric juice, which will then permit easier absorption [95]. Even so, most aglycones will be absorbed only at the intestinal level [95] and enter the enterocyte by passive diffusion. Some glycosides, however, are probably transported by active transport through a sodium-dependent glucose transporter (SGLT1) [96] and hydrolyzed afterwards in the cell by cytosolic β-glucosidases into the enterocyte.

When metabolized, phenolic compounds can give rise to numerous compounds that make their detection in vivo particularly difficult, not only because it varies from one phenolic compound to another, but also because a large number of new molecules can be produced at concentrations far below the concentration of the ingested parental compound [97]. Enterocytes have an important metabolic capacity, so polyphenols usually undergo phase I reactions of hydrolysis, reduction and oxidation, which introduces or exposes functional groups, such as the hydroxyl group, suitable for phase II metabolic reactions [97]. The presence of the phenolic ring in the structures of antioxidants, however, allows the occurrence of metabolic pathways common to all classes of phenolic compounds [97]. In most cases, the aromatic structures of the antioxidant are maintained, since the existence of free hydroxyl groups on the aromatic ring can already undergo sulfonation, glucuronidation and/or methylation conjugation reactions by the action of sulfotransferase enzymes (SULT), uridine-5-diphosphate glucuronosyltransferases (UDG) and catechol-O-methyltransferases (COMT), respectively [98]. Conjugation by sulfonation and glucuronidation leads to a marked increase in the hydrophilicity of the molecule caused by the negatively charged sulfate and glucuronide groups with a great capacity to form hydrogen bonds. This higher hydrophilia makes it difficult for these metabolites to cross cell membranes and, therefore, once produced, they require specific ATP-binding cassette transporters (ABC transporters) [99] to be sent into the bloodstream. The conjugated metabolites produced can also be sent back to the intestinal lumen via specific efflux ABC transporters. Through the hepatic portal vein, compounds released by enterocytes into the bloodstream reach the liver and can enter hepatocytes through several families of organic anion transporters (OAT), namely, the peptide family (OATP) and the multidrug resistance-associated protein (MRP) family (ABC transporters). This type of carrier is quite specific for the type of conjugated group, the phenolic class and to the position of the aromatic hydroxyl group that underwent the conjugation reaction [100].

In the liver, other phase I metabolic reactions (catalyzed by dependent and nondependent cytochromes P450 enzyme systems) and other phase II reactions (catalyzed by the UGA, SULT, COMT and glutathione-S-transferase enzymes) may occur. Metabolites are normally sent back into the bloodstream by the liver, distributed to organs and tissues, and then carried to the kidneys to be excreted in the urine. Although it is considered that the production of sulfates and glucuronides aims to facilitate the excretion of xenobiotics in the urine, their hydrophilicity allows the transport of these metabolites through the blood, reaching various tissues where they can later be deconjugated by enzymes such as β-glucuronidases or sulfatases. Several studies show that conjugated metabolites may work as temporary deposits in tissues as intracellular deconjugation is greatly increased during inflammation, and this seems to contribute, in part, to the bioactivity of many conjugated metabolites [101].

As only 10–15% of polyphenols seem to be absorbed in the small intestine [100], therefore, most reach the large intestine, where they can suffer the action of microbiota. As an important number of phenolic metabolites can be produced by the action of microbiota, at least part of the observed bioactivity related to antioxidants results from these microbiota metabolites. The microbiota has a range of enzymes that are able to hydrolyze glycosides to oxidize or reduce the non-phenolic moieties of antioxidants and to catalyze ring fissions, leading to the production of smaller phenolic compounds [102]. Usually, these metabolites are more lipophilic and able to be absorbed by the enterocyte. Afterwards, they can be subjected to further metabolism of phase I and phase II and be responsible, to a large extent, for the bioactivity of antioxidants. In the case of flavonoids, ring fusions are of particular importance because small phenolic acids with C6-C3 and C6-C1 backbone are produced, sometimes with a higher bioactivity than the parental compound [103]. The importance of the microbiota for the metabolism and bioactivity of antioxidant polyphenols has gained great interest in the scientific community, since it has been found that one of the main causes for the inter-individual variation in the bioavailability and bioactivity of polyphenols is caused by differences in microflora. In fact, these variations appear to be even more important than those observed when considering gender, age, body mass index or drug intake [104]. For example, inter-individual variations in the recovery of flavonoids and olive oil secoiridoids ranged from 2 to 60%. Since these compounds also appear to modulate the existing microbiota, the bioavailability of a given phenol may also vary with the continued consumption of certain antioxidants [104].

## 9. Nano Antioxidant Delivery Systems (NDDS): Antioxidant Bioavailability Improvement

An antioxidant candidate with perfect in vitro properties may fail in clinical trials due its low bioavailability [105]. To achieve the desired effect from an antioxidant, it must pass through multiple biofilm layers within the body and exhibit good absorption, distribution, metabolism and excretion properties. The physicochemical stability, aqueous solubility, permeability and lipophilicity of the active molecules have a crucial impact on these properties. However, since many active ingredients cannot be formulated properly, they have bioavailability problems and cannot be marketed. Therefore, tremendous attention is given to improving the in vivo pharmacokinetic and pharmacodynamic properties of pharmaceutically active agents. In many cases, conventional antioxidant formulation strategies lack specificity, stability and effectiveness and may lead to several unpleasant side effects. To overcome these restrictions, the concept of antioxidant delivery systems has been developed. As a reflection of the developments in nanotechnology to pharmaceutical R&D, studies have been carried out on nano-drug delivery systems (NDDS). A wide range of active molecules, such as drugs, biomolecules and phytochemicals, with various solubility profiles can be either conjugated or encapsulated in NDDS. These systems are well tolerated in vivo due to their biocompatible and biodegradable nature, and they can improve both the in vitro and in vivo properties of the loaded active molecule. NDDS have unique physicochemical properties due to their large surface area [106]. NDDS can circulate in the blood for a prolonged period, carry one or more active molecules, target specific tissues and improve the solubility and therapeutic efficacy of the encapsulated drug. Recently, the number of nanotechnology-based drug formulations on the market has increased. According to the statistics between 2008 and 2020, 65 percent of the ongoing clinical trials with NDDS were cancer trials [107]. Liposomes, protein-based nanoparticles, oxide/metal-based nanoparticles, polymer-based nanoparticles, lipid-based nanoparticles and micelles are the most investigated nanocarriers.

It is known that antioxidants have high potential in the effective treatment of diseases such as inflammatory diseases, digestive system diseases, neurodegenerative diseases and cancer. The concept of formulating antioxidants is a crucial issue for efficiently using them in disease treatment. Ensuring their efficacy by maintaining the antioxidant concentrations within a therapeutic window is not always possible with conventional dosage forms. Unfortunately, factors such as low aqueous solubility, weak physicochemical stability, first-pass metabolism and enzymatic instability reduce the bioavailability of most of the antioxidants [108]. This situation hinders the formulation of antioxidants as a final product and their effective use in clinical practice. Therefore, it is necessary to develop new formulation strategies, and, in parallel with this need, the approach of formulating antioxidants as nano-drugs has become attractive. Different types of NDSS have different physicochemical and morphological properties. Antioxidant components with different polarities can be loaded into NDSS through various chemical (such as covalent bonds) or physical interactions (van der Waals interactions) [109]. The major classes of NDDS utilized to deliver antioxidants are listed in Table 6 along with their advantages and disadvantages. This section focuses on the bioavailability improvement and site-specific targeted delivery of antioxidants via NDDS.

### 9.1. Targeted Delivery of Antioxidants via NDDS

**Figure 13:**
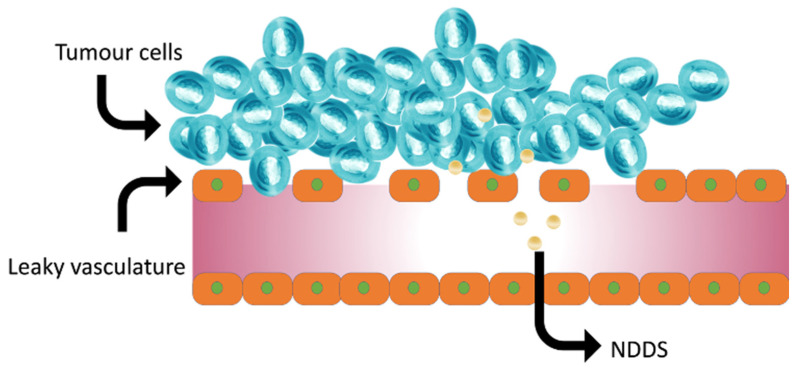
Schematic presentation of passive drug targeting and enhanced permeability and retention effect (EPR) in tumor tissue. The passive targeting of NDDS to tumors occurs through the spaces between endothelial cells, and a decrease in lymphatic drainage improves this accumulation.

Targeted drug delivery is of great interest for selectively carrying the active pharmaceutical ingredients (APIs) to the site of action for a prolonged period at a therapeutically adequate amount. In this way, drug delivery to non-target tissues is prevented and systemic/local toxicities are reduced [110]. NDDS can be targeted to specific organs/tissues by two main mechanisms, known as passive or active targeting (Figure 13). NDDS can be designed in a way so that one or both targeting strategies can be used. In the case of passive drug targeting, specific characteristics such as size or surface charge trigger the targeting process, and physiological factors also contribute to the targeting process. The deterioration of the physiological structure in various disorders and the physiochemical properties of NDDS can enable the passive accumulation of NDDS in the tissues [111]. It is known that vascular permeability is high in diseases such as cancer and rheumatoid arthritis. This leads to the accumulation of NDDS in the bloodstream in tumors or inflamed tissue. This mechanism, known as the enhanced permeability and retention effect (EPR), is the main theory for passive drug targeting [112].

**Table 6:** Advantages and disadvantages of common NDDS used to deliver antioxidants.

NDDS	Advantages	Disadvantages
**Nanoparticles:** Spherical drug carriers fabricated from synthetic or natural components in the range of 10–1000 nm [113,114]	Provide targeted drug delivery and increase retention time in the site of action Easy to prepare Reduced side effects, improve pharmacokinetic and pharmacodynamic properties of the encapsulated drug Drug loading capacity is high and does not require any chemical reaction	Some types are not biodegradable Low stability in circulation Inadequate tissue distribution and potential toxicity, especially in long-term administration
**Liposomes:** Nanosized (50–1000 nm) spherical vesicular drug delivery systems made of bilayered phospholipids in an aqueous medium [115]	Can be prepared easily and in large quantities Decompose without causing toxicity in the biological environment/Biocompatible Have cell membrane-like properties Can carry both hydrophilic and hydrophobic drugs Can be tailored as stimuli responsive systems Surface modification is possible for drug targeting	Necessity of working under special conditions (e.g.: inert atmosphere) during the preparation Short shelf-life Sedimentation, aggregation and fusion problems The purity of natural phospholipids can vary Differences between production batches Sterilization problem High production costs
**Solid Lipid Nanoparticles:** Nanocarriers (50–1000 nm) that are composed of solid lipids at body temperature. Drugs can be embedded to their core or attached to their surface [116]	Increase the bioavailability of drugs Protect unstable molecules against degradation Can be produced easily in large quantities Have stable structure Remains in the blood circulation for long time Can carry both hydrophilic and hydrophobic drugs	Low cargo capacity due to their crystalline structure High water content in their dispersions Variable drug release profiles The use of organic solvents during their preparation may cause toxicity
**Proliposomes:** Provesicular systems in dry granular form that assemble liposomes upon hydration with water or body fluids. They are composed of carrier powder, phospholipids and cholesterol [117]	More stable than conventional liposomes Can carry both hydrophilic and hydrophobic drugs Improve the bioavailability and solubility of the loaded drug Provide controlled/prolonged drug release in target tissues Reduce drug toxicity and mask the bad taste Biodegradable and biocompatible systems Easy and inexpensive to prepare Easy to sterilize Can be filled in unit dosage forms such as capsules, tablets…etc.	The purity of natural phospholipids can vary Problems (drug leakage, liposome aggregation) may arise during hydration
**Polymeric micelles:** Spherical nano-drug carriers, ranging in size from 10 to 100 nm and consist upon exposure of amphiphilic molecules to water [118]	Improve the solubility of poorly water-soluble drugs Prolonged retention time in blood circulation Accumulate in specific tissues Surface modification is possible Can be easily produced in large quantities Protect the encapsulated cargo from inactivation in the biological environment	Limited encapsulation efficiency Cannot encapsulate hydrophilic drugs Scale up problems The variety of polymers that can be used is limited
**Phytosomes:** Liposome-like lipid-based NDDS manufactured by the interaction between plant extracts and phospholipids [119]	High entrapment capacity Biocompatible systems Provide enhanced bioavailability Simple manufacturing process	Low physicochemical stability Scale up problems

**Figure 14:**
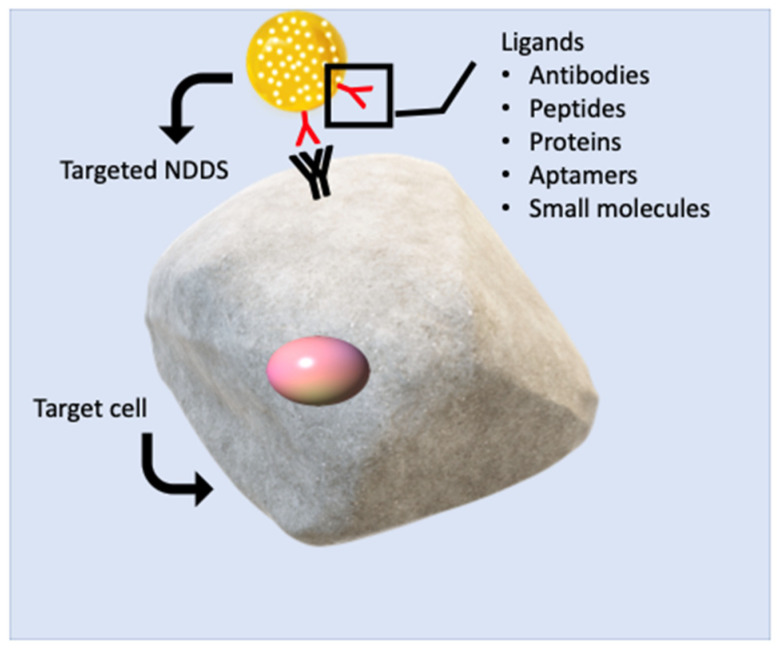
Receptor-mediated active targeting of ligand-modified NDDS. NDDS bind to the relevant specific receptor on the cell surface via the targeting ligands on their structure. NDDS, which undergo receptor-mediated endocytosis after binding, internalize into the cell and release the encapsulated active molecule in the target cell.

In passive targeting, the specificity of the NDDS to the tissues is not always at a sufficient level, and this limits the treatment’s success. To overcome this problem, NDDS modified by antigen/receptor specific ligands are developed to achieve active targeting via ligand–receptor recognition [120]. Thus, actively targeted NDDS can improve therapeutic effect and decrease toxicity in healthy tissue [121]. Important considerations in active targeting are the affinity of the ligand to the tissue and the number of receptors. Transferrin receptor, folate receptor, epidermal growth factor receptor (EGFR) and glycoproteins expressed on cell surfaces are the most studied receptors. Different targeting moieties including antibodies, peptides, proteins, aptamers and small molecules (Figure 14) are used [122,123]. Moreover, NDDS, which are stimuli (pH, temperature, ROS, etc.) sensitive, have been developed to improve site-specific delivery of drugs and provide controlled content release.

The particle size and distribution of NDDS are very critical in their biodistribution. This directly affects the extent of the drug’s retention time in the blood. NDDS larger than 200 nm can be caught in the liver, but smaller ones (less than 20 nm) are cleared by the kidney [124]. Several NDDS used for the targeted delivery of antioxidants are highlighted in Table 7. Depending on the nature of the targeted tissue, different peptides, small molecules, aptamers and antibodies are used. It was recently shown that some of the active molecules isolated from medicinal plants may also self-assemble into NDDS [125]. Natural antioxidants such as betulinic acid (BA) are among these molecules. BA-based nanoparticles were prepared by conjugating a CXCR4 antagonist, AMD3100, on the carrier’s surface. The nanoparticles were shown to reduce the infarct area in stroke mice brains and increase the survival rates due to antioxidant activity and increased drug transport to the ischemic brain [126].

### 9.2. Improved Bioavailability via NDDS

Oral administration of drugs might be problematic due to their low solubility and poor permeability. In addition, the bioavailability of some drugs is low because they are substrates of glycoprotein and cytochrome P450 3A4 (CYP3A4). This leads to high interindividual variability in terms of pharmacokinetic properties. Therefore, various techniques and strategies are developed to improve the oral bioavailability of such active agents. Chemical modification, co-crystal technology, solid dispersions, microparticle systems and NDDS are being studied for this purpose [134]. Recent studies revealed the superiority of NDDS in terms of improving oral bioavailability. As oral drug administration provides high patient compliance and a lower risk of complications, the approach of nanotechnology-based oral drug delivery is growing.

**Figure 15:**
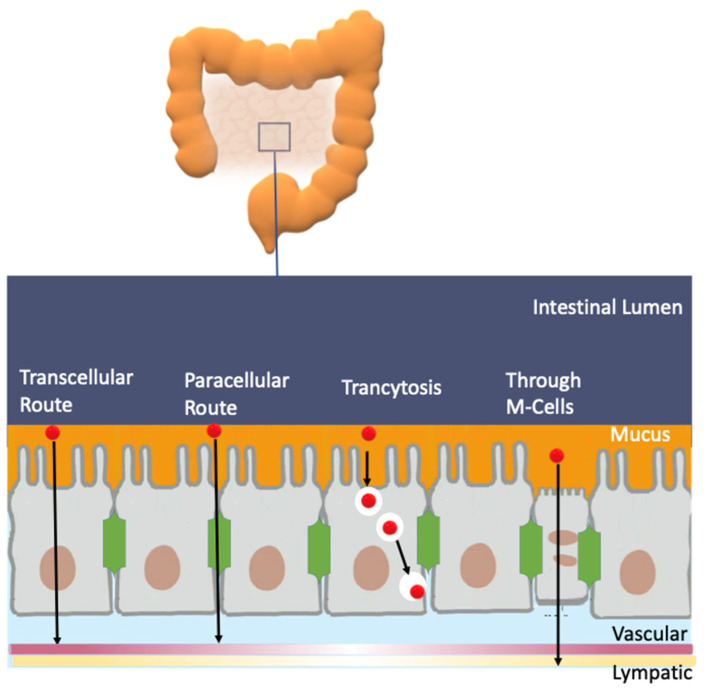
Schematic illustration of intestinal NDDS absorption. Transport of free drugs is inhibited via the epithelial cell membrane, viscous mucus layer and intercellular tight junction. Gastrointestinal enzymes and ABC efflux pumps also decrease the intestinal absorption. Possible absorption pathways for NDDS are shown by arrows.

It has been shown that NDDS can increase the oral bioavailability of active molecules by different mechanisms. They may increase the solubility of the active substance, alter the absorption pathways, protect them from enzymatic degradation in the gastrointestinal (GI) tract, inhibit the ATP-binding cassette sub-family B member 1 (ABCB1) efflux transporter and the cytochrome P450 enzyme system and can be targeted to specific regions of the GI tract [135]. There are different pathways for the passage of NDDS across the intestinal membrane (Figure 15). In the absence of targeting moieties on the cell surface, the NDDS can be absorbed intact by enterocytes and M cells via an endocytotic pathway triggered by non-specific interactions such as hydrogen bonding or van der Waals interactions. NDDS can be absorbed by pinocytosis, where the cell surface captures the carrier. Furthermore, they may be absorbed by the receptor-mediated endocytic route, an approach that has been widely studied in parenteral drug delivery but rarely investigated in oral drug administration [136].

A wide range of active molecules with antioxidant activity was formulated in NDDS to improve their bioavailability (Table 8). The success of different types of NDSSs examined in in vivo, ex vivo and in cell culture studies in improving the efficacy of orally administered antioxidants has been demonstrated. The activity considerably depends on the NDSS’ composition, encapsulation efficiency, surface charge, particle size and distribution. Regarding the size of NDDS, studies revealed that smaller particles tend to pass through the intestinal barrier via transcytosis with a higher efficiency compared to larger particles. Furthermore, as the intestinal epithelial cells possess a negative charge, positively charged NDDS can be electrostatically attracted, and intestinal absorption may increase [137,138]. Curcumin is one of the most promising natural antioxidants, but its clinical usage is limited due to poor physicochemical characteristics. Curcumin-encapsulating NDDS (γ-cyclodextrin, phytosome, liposome, microemulsion and solid dispersion) were examined in human subjects for their potential on improving curcumin oral bioavailability. The review article on this subject by Pan-on et al. clearly emphasizes the success of NDDS in improving curcumin’s bioavailability by enhancing aqueous solubility and stability [139].

The outcome of the reviewed studies has highlighted that the use of NDSS in the formulation of bioactive molecules resulted in better biological activity compared to the unformulated compounds. NDDS can obviously potentiate the beneficial effects of antioxidants. Despite the positive findings obtained in the studies, clinical use of NDSS is still a subject requiring further research. NDSSs must possess repeatable standards in terms of critical quality properties such as loading capacity, drug release kinetics and stability. The scaling-up of the manufacturing process, long-term toxicity and biocompatibility studies are several vital points to consider for designing highly effective NDSS.

## 10. Antioxidants in Diseases: General Evidence

### 10.1. Effects on Cardiovascular Diseases, CVDs

Cardiovascular diseases (CVDs) include several disorders associated with the blood vessels and heart such as atherosclerosis, stroke, hypertension, cerebrovascular and peripheral arterial diseases. The evolution of a number of these disorders can be inhibited or delayed by preventing different risk parameters such as hypertension, tobacco, obesity, lack of physical activity and unhealthy dietary habits.

**Figure 16:**
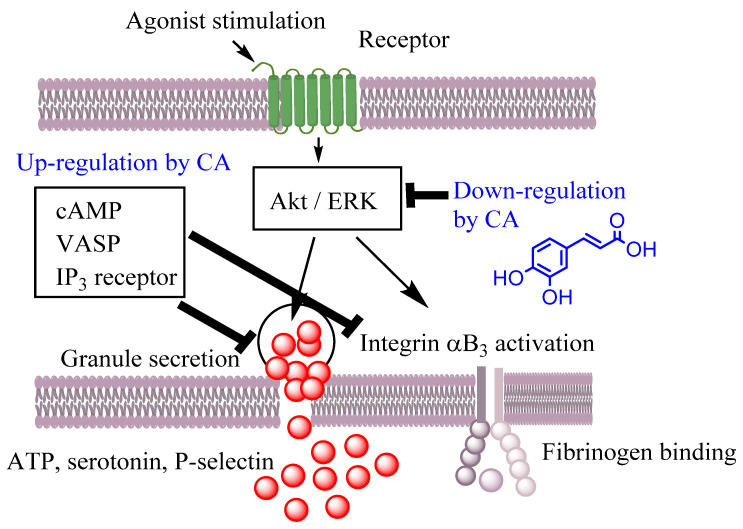
Effects of caffeic acid (CA) on platelet activation in thrombin-simulated platelets (adapted from [148]). Caffeic acid may act as a potential therapeutic compound by inhibiting thrombin-induced platelet aggregation, adenosine 1,4,5-tri-phosphate (ATP) release, the phosphorylations of protein kinase B (AKt) and extracellular signal-regulated kinase (ERK). Moreover, CA may also improve cyclic adenosine monophosphate (cAMP) production, which may induce the phosphorylation of the vasodilator-stimulated phosphoprotein (VASP) and inositol trisphosphate (IP3) receptor. Reprinted from [148], Copyright (2022), with permission from Elsevier.

Several studies have showed a relationship between the mitigation of the progression of these diseases and the intake of polyphenol rich fruits and vegetables [145]. It was hypothesized in different studies that the presence AOs may decrease blood pressure and glucotoxicity, ameliorate insulin resistance and pancreatic beta cells’ activity and prevent platelet aggregation, among other actions indicated below [145] (Figure 16).

Calabriso et al. showed that hydroxytyrosol (HT) can present an endothelial protective effect by decreasing the formation of superoxide anion in the mitochondria of PMA-activated endothelial cells, reducing membrane lipid oxidation by promoting SOD activity. Likewise, HT enhances mitochondrial biogenesis by increasing mtDNA content and peroxisome proliferator-activated receptor gamma coactivator-1α (PGC-1α) expression, mitochondrial transcription factors A (TFAM) gene expression and the transcription factor nuclear respiratory factor-1 (NRF-1) [146]. Song, J. et al. [147] found that resveratrol reduces intracellular reactive oxygen species levels by inducing autophagy through the AMPK-mTOR pathway, promoting cardioprotection in metabolic syndrome. Caffeic acid has also been found to be a potential therapeutic agent for thrombotic disorders. Nam et al. found that caffeic acid inhibited thrombin-induced platelet aggregation, delayed the kinetics of clot retraction and inhibited fibrinogen binding to integrin αIIbβ3 with this antiplatelet effect, associated with a cAMP-dependent pathway, Figure 16 [148].

AOs also have the ability to provide various beneficial effects in vivo. In an in vivo study, Cai Y. et al. [149] reported that epigallocatechin-3-gallate (EGCG) may enhance Nmnat2 protein expression and enzyme activity in cultured neonatal rat cardiomyocytes and rats with abdominal aortic constriction. Similarly, another study reported the influence of kaempferol and kaempferol nanoparticles on 5-FU-induced cardiotoxicity in rats, indicating that their administration decreases oxidative damage, COX-2 and VEGF expression and cardiac enzymes [150]. Additionally, protocatechuic acid has been found to decrease the concentration of cholesterol and to attenuate hepatic lipid deposition in rats with coronary artery disease produced by a high fat and fructose diet [151]. Gallic acid has also showed cardiovascular-protective effects in hypertensive rats [152]. The administration of gallic acid decreased the transcript levels of cardiac Nox1, Nox2 and Nox4 and attenuated the expression of Nox protein. Another study conducted with rats showed that, in rats with isoproterenol-induced myocardial infarction, the oral administration of ellagic acid for a period of 10 days was able to reduce lipid damage, pathological arrhythmias, ventricular hypertrophy, myocardial necrosis and dyslipidemia [153]. In a recent study, the supplementation of delphinidin-3-O-glucoside was shown to decrease the inflammation and oxidative damage in induced atherosclerosis by a high fat diet in a rabbit model, reducing lipid content in the liver and aorta [154].

Several studies about the cardiovascular protective effect of antioxidants have also been performed in humans. Quirós-Fernández et al. reported the beneficial effect of hydroxytyrosol supplementation together with punicalagin on atherosclerosis markers in patients aged between 45 and 65 years [155]. Salazar et al. [156] found, in a group of middle-aged asymptomatic patients, a negative relationship between dietary supplementation of different antioxidants and plaques in the femoral and carotid arties. A lower incidence of coronary calcium and subclinical atheroma plaques was also observed, decreasing cardiovascular risk.

Several human experimental studies with curcumin supplementation have shown cardiopreventive effects by decreasing low density cholesterol (LDL-C), total cholesterol, triglycerides, lipoprotein a (Lp(a)), serum lipid peroxides and the TC/HDL-C ratio, among other parameters [157,158].

### 10.2. Effects on Neurodegenerative Diseases

The consumption of antioxidant-rich diets was shown to alleviate the harmful impact of neurodegenerative diseases (Alzheimer’s disease, Parkinson’s disease, depression multiple sclerosis, Huntington’s disease, etc.), probably due to the potential neuroprotective activity of polyphenols through the activation of signaling pathways related to protein kinases such as Keap1/Nrf-2/ARE, the main protective pathway against ROS damage [159].

Ratman et al. reported the neuroprotective effects of resveratrol by employing different models of Alzheimer’s disease, both in vivo and in vitro [160]. Its neuroprotective effect is caused by the decrease in intracellular calcium and ROS synthesis in primary cortical neurons, the decrease in the NO generation in hippocampal cells, inhibition of NO synthesis and expression of iNOS and COX-2 in rat astroglioma C6 cells and reduced PGE2 and PGD2 synthesis due to COX-2 inhibition in SK-N-SH cells. Moreover, resveratrol combined with melatonin was shown to reestablish the cognitive deficits and to reduce the oxidative damage and reduce BDNF expression in a rat model of vascular dementia [161].

The beneficial health effects of tea polyphenols, such as epigallocatechin-3-gallate (EGCG), were widely reported both in vivo and in vitro. EGCG was shown to decrease the level of beta-amyloid plaques in Alzheimer transgenic mice [162], inhibit neuronal apoptosis [163] and enhance neuronal survival and hippocampal neurogenesis [164].

Quercetin has been shown to ameliorate cognitive dysfunction at the early stage of development of Alzheimer’s disease, and the protective effect was mainly related to increased amyloid-beta peptide (Aβ) clearance and reduced astrogliosis [165]. In another study, quercetin was also shown to ameliorate Aβ toxicity in a Drosophila AD model by modulating cell cycle-related protein expression [166].

Curcumin has also been reported for its neuroprotective effect by counteracting the reduction in the enzyme tyrosine hydroxylase, hypothesized as a key factor in the development of Parkinson’s disease [167]. In addition, the supplementation of curcumin in rats with oxidative stress-induced injury of Parkinson’s disease was shown to increase viability, survival and adhesion and decrease the apoptosis of deutocerebrum primary cells through activation of the Wnt/β-catenin signaling pathway [168].

The dietary administration of hydroxytyrosol was shown to promote the activity of mitochondria and reestablish the deficit of energy in the 7PA2 cell model of Alzheimer’s disease [169]. Likewise, González-Correa et al. [170] reported that the supplementation of hydroxytyrosol also reduces the number of retinal ganglion cells in streptozotocin-diabetic rats. Another study [171] showed that hydroxytyrosol can activate neurogenesis in both aged wild-type mice and B-cell translocation 1 gene knockout mice model of accelerated neural aging by reducing apoptosis and enhancing the survival of new neurons. Furthermore, oral administration of extra virgin olive oil or hydroxytyrol has been shown to reduce the degree of lipid and protein oxidation, increase glutathione peroxidase and reduce both bacterial lipopolysaccharide (LPS) and LPS-binding protein (LBP) products of the microbiota in the brain, spinal cord and blood in an induced rat model of multiple sclerosis [75].

### 10.3. Effects on Digestive Diseases

Various diseases can affect the digestive tract, including inflammatory disorders such as Crohn’s disease (or ulcerative colitis) and peptic ulcer disease [172]. The administration of resveratrol is shown to reduce MDA levels, enhance glutathione (GSH) content and the activity of catalase and to decrease the inflammatory cytokines levels in the intestine of irradiated rats [173]. Resveratrol is also able to reduce inflammatory cytokines levels in colitis [174] and to reduce the ulcerative area and index of colon mass in a trinitrobenzene sulfonic acid (TNBS)-induced ulcerative-colitis model [173]. Likewise, the dietary supplementation of quercetin alleviates experimental colitis in mice by controlling the bactericidal activity and anti-inflammatory activity of macrophages through a heme oxygenase-1 dependent pathway [175]. Another study has reported that the administration of caffeic acid also ameliorates colon inflammation and oxidative damage, probably by modulating gut microbiota in mice [176]. Chlorogenic acid has also been shown to ameliorate colon mucosal injury in rats fed with a high fat diet [176]. S. Larrosa et al. investigated the combined effects of hydrocaffeic, dihydroxyphenylacetic and hydroferulic acid metabolites from colon microbiota and found that these polyphenols can reduce the synthesis of prostaglandin E2 in IL-1β-stimulated CCD-18 colon fibroblast cells [177]. Gallic acid has also shown beneficial effects on the induced gastric mucosal damage by inhibiting the oxidative damage and reducing apoptosis in gastric mucosal cells [178]. Curcumin is another antioxidant that may have beneficial effects in inflammatory bowel disease and mucosal inflammation by interacting with many cellular targets (NF-κB, JAKs/STATs, MAPKs, TNF-γ, IL-6, PPARγ and TRPV1) [179].

### 10.4. Effects on Cancer

Many epidemiological studies have been published revealing the preventive properties of polyphenols by employing different in vitro and in vivo models. Studies have shown how the treatment with polyphenols could promote apoptotic cell death in preneoplastic or neoplastic cells through a variety of mechanisms, including the modulation of cell cycle signaling, antioxidant enzyme activity and arrest of the cell cycle, among others, mitigating the development of tumors. Recent in vitro studies have supported that these compounds can moderate Nrf2 and NF κB activation in cells and considerably affect MAPK and PI3K function in cells, showing their influence in the propagation of cancer cells [180]. Phenolic compounds affect carcinogenesis by the modulation of cell defense systems, including antioxidant enzymes and anti-inflammatory and anti-cellular growth signaling pathways that will trigger cell death. These observations clearly support the anticancer effects of polyphenols by changes in the cancer cells’ epigenome [180].

In vitro and vivo studies have shown the chemopreventive effect of quercetin in oncology. Dietary supplementation with quercetin has been shown to mitigate the cell viability of colon cancer cell lines HCT-15 and RKO and modulate the breast cancer cell lines MCF-7, MDA-MB-231, HBL100 and BT549 and the ovarian cancer cell lines OVCAR5, TOV112D, OVCAR3 and CAOV3 [181].

Caffeic acid phenethyl ester has been shown to modulate cell cycle control genes in breast cancer cell line MCF-7 [182]. Likewise, in a recent study, chlorogenic acid caused apoptosis and reduced metastasis via the NF-κB signaling pathway in breast cancer [183]. Hydroxytyrosol has also been shown to cause apoptosis in colorectal cancer cells by increasing the expression of the CASP3 gene and antioxidant enzyme activity and by inhibiting the development of LS180 cells through the modification of the antioxidant defense system [184].

Furthermore, epigallocatechin-3-gallate (EGCG) has also proven to have a chemoprotective effect on pancreatic cancer cells. EGCG impacts glycolysis by the inhibition of extracellular acidification via the inhibition of both the activity and content of the glycolytic enzymes [185].

Curcumin treatment has also been shown to promote apoptosis, the down-regulation of ERK and cell-cycle arrest in phase S and the inhibition of the p-ERK-2/c-Jun pathway in model cells of endometrial carcinoma [186].

According to the information available at ClinicalTrials.gov (accessed on 11 April 2022), various clinical trials have reported the anti-cancer effect of different antioxidants. However, most of them, although finished, do not yet have available findings [181]. For example, for quercetin, there are only three complete studies; two of these are about its influence in prostate tissue from patients with prostate cancer (NCT01912820 and NCT03493997) and the third is about the prevention and treatment of chemotherapy-induced oral mucositis in blood dyscrasias (NCT01732393). In the case of hydroxytyrosol, there is only one active clinical trial (NCT02068092) about its effect on breast cancer. In the case of resveratrol, there are five completed studies but with no results reported, and one clinical trial concluded with results reported in the ClinicalTrials.gov (accessed on 11 April 2022) database, in which its effects in patients with colon and liver cancer, multiple myeloma and low grade gastrointestinal tumors were analyzed. For caffeic acid, there is an active study in phase 3 investigating its efficiency in Chinese advanced esophageal squamous cell cancer. For chlorogenic acid, there are one completed (NCT02245204) and one finished (NCT02136342) studies in patients with advanced cancer and another completed study (NCT02728349) in patients with advanced glioblastoma. These studies reveal the need of carrying out more research in different clinical phases in order to support the preclinical evidence about the pharmacological activity of polyphenols, evaluating both their efficiency and safety for the prevention and treatment of various kinds of cancer.

### 10.5. Effects on Inflammation

Inflammation is a comprehensive array of physiological responses to foreign organisms, such as human pathogens, dust particles and viruses. Inflammation can appear in patients with infections, immune diseases and chronic diseases such as diabetes, rheumatoid arthritis and cancer. Polyphenols possess the capacity to modulate the inflammatory routes of usual arthropathies such as osteoarthritis and rheumatoid arthritis. The health effects of the consumption of quercetin, resveratrol, EGCG, p-coumaric acid, curcumin, kaempferol, etc. through a diet rich in fruits and vegetables and their effect on arthritis and other inflammatory diseases have been extensively studied [3].

Resveratrol has shown anti-rheumatoid arthritis properties by decreasing rheumatoid arthritis patients’ swelling, tenderness and disease activity via decreasing the biochemical markers of inflammation such as MMP-3, ESR, C-reactive protein, IL-6 and undercarboxylated osteocalcin [3]. Likewise, in another study, resveratrol has also been shown to minimize inflammation and restore small airways in the lung tissue of a rat model of chronic obstructive pulmonary disease [187].

Curcumin was found to modulate NF-κB (nuclear factor kappa-light-chain-enhancer of activated B cells) and STAT3 (signal transducer and activator of transcription) and to inhibit the expression of TLR-2 (toll-like receptor-2) and 4 while, in vivo, it modulates PPARγ (Peroxisome proliferator-activated receptor gamma) in male adult rats [188].

Quercetin has been proven to inhibit the biosynthesis of leukotrienes in human polymorphonuclear leukocytes [188]. Similarly, EGCG mitigated the activation of NFκ B in human epithelial cells and modulated the expression of iNOS (inducible nitric oxide synthase) and the formation of NO (nitric oxide) in macrophages, leading to its immunomodulation [188].

Many remarkable findings have also been reported concerning the beneficial effects of polyphenols on SARS-CoV-2 illness [189]. Treatment with resveratrol, curcumin, EGCG and quercetin, among other polyphenols, could exert an inhibitory effect on the angiotensin-converting enzyme 2 receptor (ACE2r)-viral spike protein linkage and on viral proteases. Likewise, based on the literature, various clinical trials are ongoing to test such hypotheses. It was reported that quercetin was shown to exert anti-inflammatory and immunomodulatory properties, being a good candidate as a therapeutic agent against COVID-19 [189]. At present, there are six completed clinical trials evaluating the protective effects of quercetin in patients with COVID-19 (NCT04578158, NCT04377789, NCT05037240, NCT04603690, NCT05037240, NCT04861298).

## Figures and Tables

**Table 1:** Rate constants for alternate reactions of peroxyl (ROO^•^) and alkoxyl (RO^•^) radicals competing with H abstraction.Data extracted from reference [11].

	Solvent	ROO^•^	RO^•^
H abstraction, LH	Nonpolar organic	<1–400 M^−1^s^−1^	10^4^–10^7^ M^−1^s^−1^
H abstraction, LOOH	Nonpolar organic	600 M^−1^s^−1^	2.5 × 10^8^ M^−1^s^−1^
Cyclization	Nonpolar organic	10^1^–10^3^ s^−1^	10^4^–10^5^ s^−1^
Addition	Nonpolar organic	—	10^4^–10^8^ M^−1^s^−1^
β-scission	Oleate Linoleate	1–8 s^−1^ 27–430 s^−1^	10^3^–10^5^ s^−1^ 10^4^–10^5^ s^−1^

**Table 2:** Inhibition rate constants (*k*_inh_) for the reaction between different ArOH and polystyrene peroxyl radicals. Data from ref. [33]. Adapted from reference [10].

	R_1_	R_2_	R_3_	R_4_	R_5_	10^−4^ *k_inh_* (M^−1^ s^−1^)
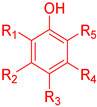 **Phenols**	H	H	CH_3_	H	H	0.917
	H	H	OCH_3_	H	H	4.78
	C(CH_3_)_3_	H	OCH_3_	H	C(CH_3_)_3_	11
	CH_3_	H	OCH_3_	H	CH_3_	94
	CH_3_	H	CH_3_	H	CH_3_	8.5
	CH_3_	CH_3_	CH_3_	H	CH_3_	11
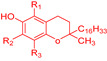 **Tocopherols**	CH_3_	CH_3_	CH_3_	—	—	320
	CH_3_	H	CH_3_	—	—	130
	H	H	CH_3_	—	—	44
	H	CH_3_	CH_3_	—	—	140

**Table 3:** The dielectric constants of some common solvents and cosolvents [46].

Solvent	Dielectric Constant ∈
Water	78.5
Ethanol	24.3
Propylene glycol	32
Olive oil	3-0
Sunflower	3.1
Corn	3.1

**Table 4:** Main effects of changes in the solvent phase that affect partitioning.

Modifications of the solvent phase	Cosolvents	Modification of the dielectric constant
	Hydrotopes	Formation of complexes, modification solvent structure, and/or self-agregation to form “micelle-like” aggregates
	Micelles	Creation of one or more regions with different solvent properties
	Liposomes	
	Microemulsions	
	Emulsions	
	Complexation	Formation of host-guest complexes
	Combined methods	Simultaneous variation in one or more experimental parameters (pH, T, cosolvent, etc.)

**Table 5:** Permeability coefficients (Pm) of some reactive species.

Molecule	Membrane	T (°C)	Pm (cm/s)	Reference
O_2_	CHO cells	37	42	[64]
NO^•^	RBC (human)	20	18	[65]
NO_2_^•^	EYPC	25	~5	[66]
H_2_O_2_	PC12 cells HUVEC cells IMR-90 cells	-	4 × 10^−4^ 1.6 × 10^−3^ 1.1 × 10^−3^	[67]
HOO^•^	EYPC	23	4.9 × 10^−4^	[68]
O_2_^−^	EYPC	23	7.6 × 10^−8^	[68]

CHO = Chinese hamster ovary cells, RBC= red blood cells, EYPC = vesicles of egg yolk phosphatidylcholine, PC12 cells = typical neuronal cell model, HUVEC cells = endothelial cell line, IMR-90 cells = cell line from fibroblasts isolated from normal lung tissue, EYPC = liposomes of egg yolk phosphatidylcholine.

**Table 7:** Recent studies on NDDS based targeted delivery of antioxidants.

Antioxidant	Target	Carrier	Targeting Ligand	Result	Reference
Vitexin	Goblet cell	Soy peptide nanoparticles	Goblet cell targeting peptide CSKSSDYQC (CSK) coupled N-trimethyl chitosan (TMC)	Improved stability in the stomach Sustained vitexin release in the intestine Absorption through tight junctions Increased bioavailability and antioxidant activity	[127]
Resveratrol	MCF-7 cells	Lipoprotein-mimetic nanoparticles	Folic Acid (FA)	Improved cytotoxicity against FA receptor-rich MCF-7 cells Improved pharmacokinetic parameters upon intravenous administration in rats	[128]
Genistein	CD44 expressed breast tumor	Phytosomes	Hyaluronic acid (HA)	Improved chemotherapeutic effect in terms of tumor size and tumor biomarkers reduction in breast carcinoma model of mice	[129]
Plumbagin Genistein	Prostate cancer cells	Liposomes	PSMA specific antibodies	Improved anticancer effect on LNCaP cells The decrease in cancer cell proliferation was attributed to the inhibition of Glut-1 transporter and Akt-3 expressions	[130]
Quercetin	Triple negative breast cancer cells	pH sensitive nickel oxide nanoparticle	Folic Acid	The FA receptor targeted nanoparticles coated with pH sensitive polydopamine provided controlled quercetin release and were very cytotoxic against MDA-MB-231 breast cancer cells	[131]
Epigallocatechin gallate (EGCG)	CD36 receptor of macrophages	Nanoparticles	KOdiA-PC	Nanoparticles targeted mouse peritoneal macrophages and reduced the inflammatory factor released and the atherosclerotic lesion of aortic arches	[132]
Rutin	Cell surface receptors of AGEs (RAGE)	Nanoparticles	Argpyrimidine (Advanced Glycation End Product-AGE)	Nanoparticles target rutin to cells via AGE and diabetogenic effects were well controlled upon its intravenous administration to diabetic rats	[133]

**Table 8:** Selected studies on NDDS-based formulations for improving the oral bioavailability of antioxidants.

Antioxidant	Study Method	Formulation	Particle Size (nm)	Findings	Reference
Catechin	Ex vivo intestinal permeation study Pharmacokinetic studies in rats	Chitosan coated liposomes (Chitosomes)	137 ± 0.82	Chitosan coated liposomes were stable against bile salts. Improved Catechin absorption was observed in uninverted rat intestine sac model. Pharmacokinetic studies on Wistar rats revealed an increase in AUC and Cmax values, thus increasing bioavailability.	[140]
Hesperidin	Ex vivo everted gut sac permeation study Pharmacokinetic, pharmacodynamic and histopathological studies in rats	Solid lipid nanoparticles	175.3 ± 3.6	~5 times higher apparent permeability coefficient was obtained compared to free hesperidin. Increased oral bioavailability due to improved solubility and permeation was observed. Due to effective suppression of oxidative stress and apoptosis, the cardiotoxicity caused by doxorubicin was reduced.	[141]
Liquiritin	Pharmacokinetic studies in rats Pharmacodynamic and histopathological studies in diabetic mice	Liposomes	91.84 ± 1.85	The solubility of liquiritin was improved via liposomes, and its oral bioavailability was 8.8 times higher than the free drug. In diabetic mice, improved hypoglycemic effect was observed due to antioxidant activity, and histopathological studies revealed the repairing ability of liposomal liquiritin to the organ damage.	[142]
Berberine	Oral bioavailability studies in rats Determination of serum cholesterol in endogenous hyperlipidemic mice	Proliposomes	116.6 ± 5.8 *	Proliposomes were suggested as solid templates for liposomes. Reconstituted liposomes increased oral berberine bioavailability ~6 times in rats. The serum cholesterol levels significantly decreased in hyperlipidemic mice.	[143]
Myricetin	Pharmacokinetic studies in rats Antioxidant and hepatoprotective activity evaluation in mice	Proliposomes	33.17 ± 0.32 *	Vitamin E TPGS-conjugated liposomes increased the oral myricetin bioavailability via facilitated mucoadhesion, permeation and controlled drug release. Further, pharmacodynamic studies revealed better antioxidant and hepatoprotective effects in CCl4 induced hepatotoxicity mice.	[144]

* Particle size of the liposomes derived from proliposomes.

## Data Availability

Not applicable.
